# Plasma exosomes improve peripheral neuropathy via miR-20b-3p/Stat3 in type I diabetic rats

**DOI:** 10.1186/s12951-023-02222-5

**Published:** 2023-11-24

**Authors:** Jiayang Li, Guangzhi Wu, Weiye Li, Xiongyao Zhou, Weizhen Li, Xiong Xu, Ke Xu, Rangjuan Cao, Shusen Cui

**Affiliations:** 1 Department of Hand and Foot Surgery, China-Japan Union Hospital of Jilin University, Changchun, China; 2 Key Laboratory of Peripheral Nerve Injury and Regeneration of Jilin Province, Changchun, China; 3 Department of Hand and Foot Surgery, The Third Bethune Hospital of Jilin University, Changchun, China

**Keywords:** Diabetic peripheral neuropathy, Plasma exosomes, miRNAs, Autophagy

## Abstract

**Background:**

Diabetic peripheral neuropathy (DPN) is one of the most common complications of diabetes and the main cause of non-traumatic amputation, with no ideal treatment. Multiple cell-derived exosomes have been reported to improve the progression of DPN. Blood therapy is thought to have a powerful repairing effect. However, whether it could also improve DPN remains unclear.

**Results:**

In this study, we found that microRNA (miRNA) expression in plasma-derived exosomes of healthy rats (hplasma-exos) was significantly different from that of age-matched DPN rats. By injection of hplasma-exos into DPN rats, the mechanical sensitivity of DPN rats was decreased, the thermal sensitivity and motor ability were increased, and the nerve conduction speed was accelerated. Histological analysis showed myelin regeneration of the sciatic nerve, increased intraepidermal nerve fibers, distal local blood perfusion, and enhanced neuromuscular junction and muscle spindle innervation after hplasma-exos administration. Compared with plasma exosomes in DPN, miR-20b-3p was specifically enriched in exosomes of healthy plasma and was found to be re-upregulated in the sciatic nerve of DPN rats after hplasma-exos treatment. Moreover, miR-20b-3p agomir improved DPN symptoms to a level similar to hplasma-exos, both of which also alleviated autophagy impairment induced by high glucose in Schwann cells. Mechanistic studies found that miR-20b-3p targeted Stat3 and consequently reduced the amount of p-Stat3, which then negatively regulated autophagy processes and contributed to DPN improvement.

**Conclusions:**

This study demonstrated that miRNA of plasma exosomes was different between DPN and age-matched healthy rats. MiR-20b-3p was enriched in hplasma-exos, and both of them could alleviated DPN symptoms. MiR-20b-3p regulated autophagy of Schwann cells in pathological states by targeting Stat3 and thereby inhibited the progression of DPN.

**Graphical Abstract:**

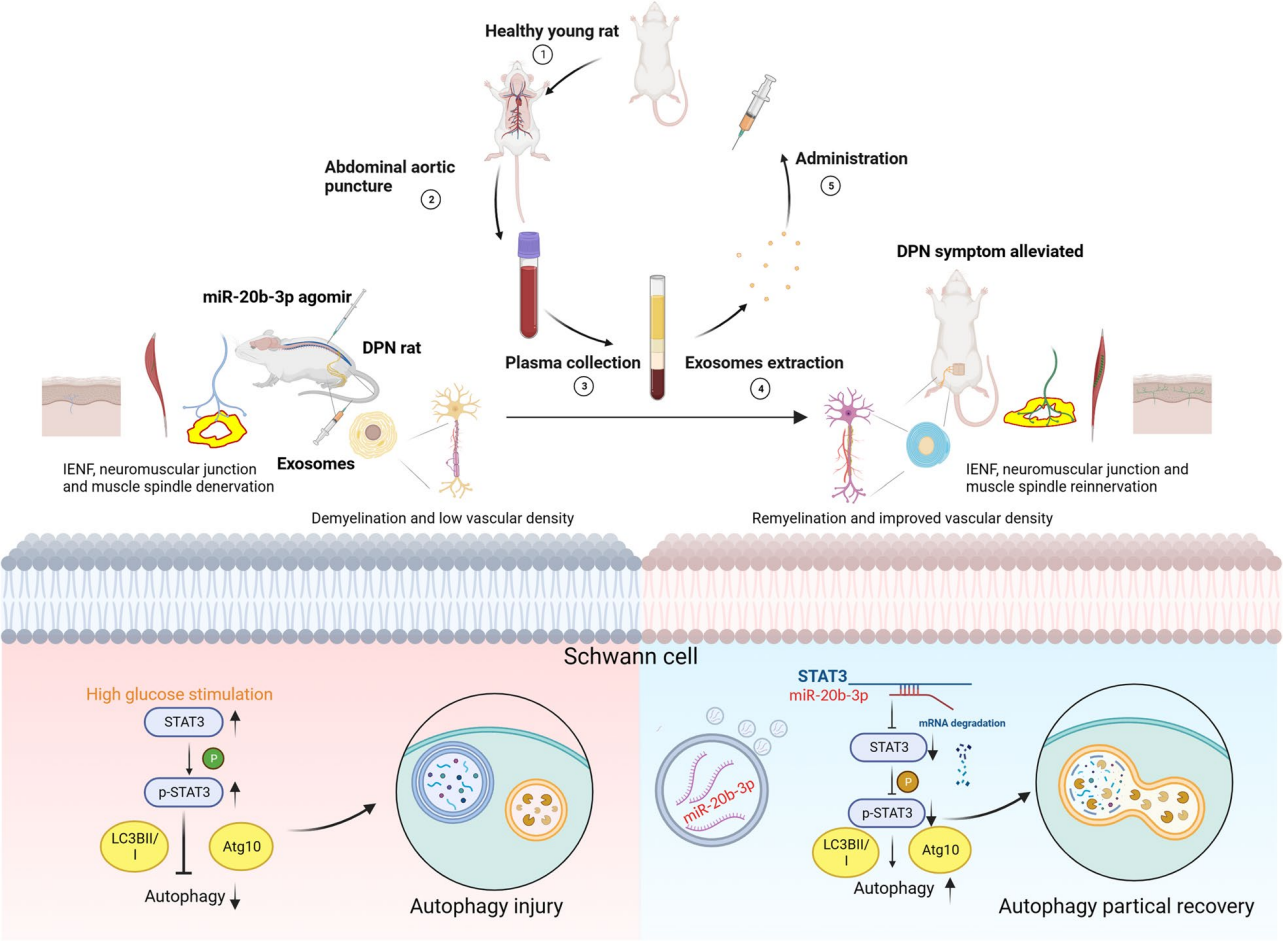

**Supplementary Information:**

The online version contains supplementary material available at 10.1186/s12951-023-02222-5.

## Introduction

Diabetic peripheral neuropathy (DPN) is one of the most common complications of diabetes. The global prevalence of diabetes was close to 500 million in 2019 and is expected to increase by 51% by 2045 [1]. The incidence of DPN is high, and nearly 50% of people with diabetes will develop DPN [2]. Patients with DPN may have symmetrical paresthesia, such as sensory loss and hyperalgesia [3]. Others have motor dysfunction, such as an increased risk of tumble, altered gait and balance, and increased body swing [4]. The pathogenesis of DPN is complex and includes axonal degeneration, nerve fiber segmental demyelination or myelin hyperplasia, and microvascular disease [5]. However, there is still has no ideal treatment for DPN [6]. Therefore, an effective treatment for DPN is urgently needed.

Exosomes are extracellular lipid bilayer vesicles with a diameter of 30–200 nm that are secreted by almost all cell types under physiological and pathological conditions [7]. MiRNAs in exosomes can be transferred to nearby or distant cells for subsequent signaling [8]. Studies have shown that mesenchymal stem cells and Schwann cell-derived exosomes improve the symptoms of DPN [9–11]. However, it is difficult to obtain materials.

Blood therapy is considered as a promising treatment. Heterochronic parabiosis therapy to exchange blood to improve ageing has been widely reported [12–14]. Studies have shown that exposure to young blood can effectively improve the tissue microenvironment in older individuals [15], and free B2M in the blood improves synaptic and cognitive function in Down’s mice [16]. A Pittsburgh study showed that extracellular vesicles in the blood carry “longevity proteins” to muscles throughout the body for repair, which suggests that extracellular vesicles are the key to return to youth [17]. During the progression of diabetes mellitus, the expression of miRNA in exosomes derived from multiple cells changes significantly [18]. Therefore, we hypothesize that plasma-derived exosomes (plasma-exos) of DPN rats themselves are quite different from those of healthy rats and aim to elucidate whether healthy plasma-derived exosomes (hplasma-exos) could alleviate peripheral neuropathy and its underlying mechanisms. Our study suggests a novel and effective intervention strategy for DPN.

## Results

### Plasma exosomes improve nerve function in DPN rats

**Fig. 1:**
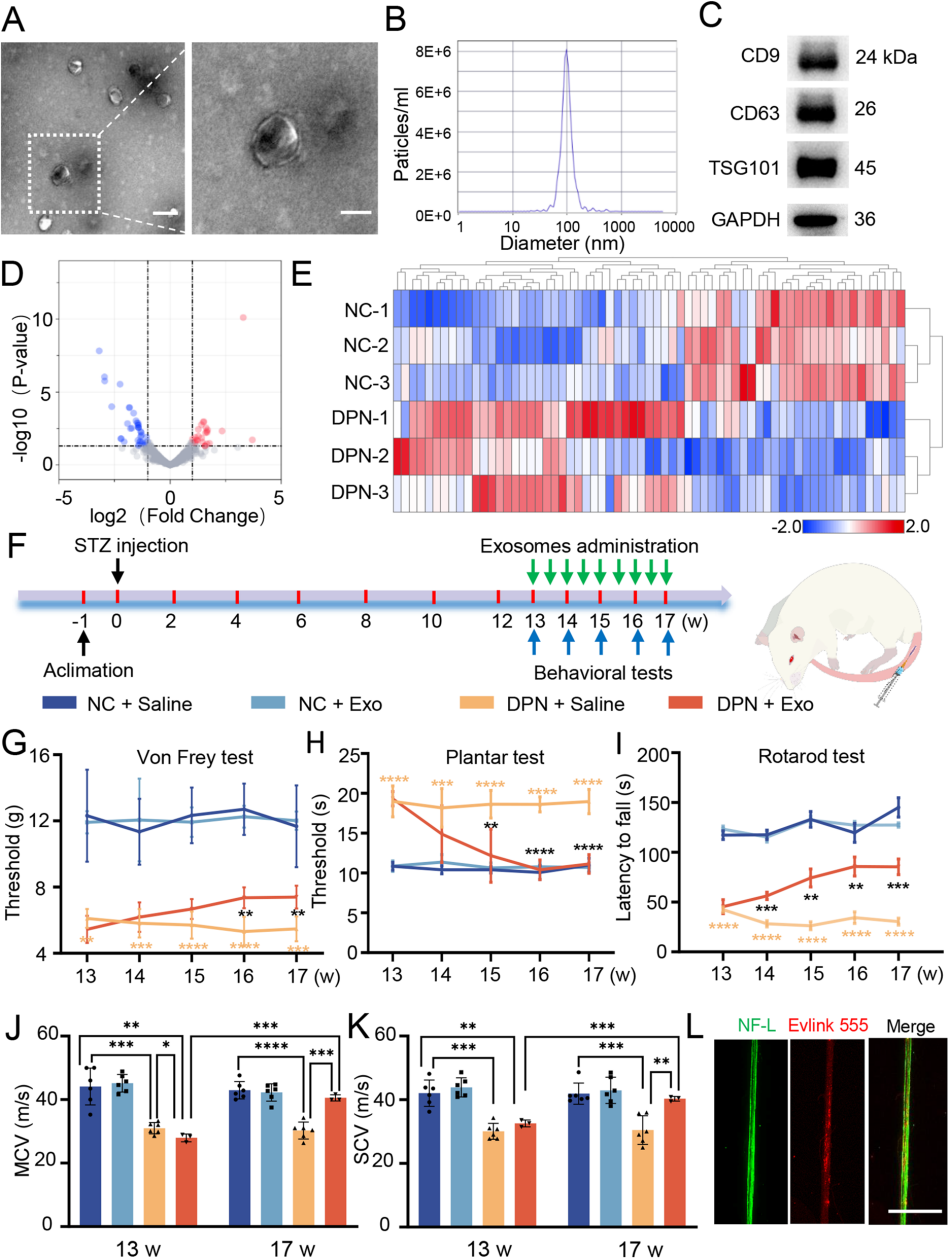
Healthy plasma-derived exosomes improve nerve function in DPN. **A** Representative TEM image of healthy plasma-derived exosomes and local magnification image, scale bar = 100/50 nm. Squares, images enlarged in right panel. **B** NTA and Western blot (**C**) were used to characterize the extracted exosomes. **D** Volcanic map and heatmap (**E**) of miRNA sequencing from plasma-exosomes in healthy and age-matched DPN rats. **F** A schematic showing the process of hplasma-exos injection. **G**–**I** The threshold of Von Frey test, plantar test and rotarod test in normal and age-matched DPN rats receiving saline or hplasma-exos treatment, n = 5. **J**, **K** Changes of MCV and SCV before and after treatment of saline or hplasma-exos, n = 3, 6. **L** The red labeled exosomes are internalized by the green labeled sciatic nerve, scale bar = 500 μm. Data are presented as the mean ± SD. (*p < 0.05, **p < 0.01, ***p < 0.001, ****p < 0.0001)

 To determine whether there are differences between DPN plasma-exos and hplasma-exos, we first established type I diabetic rats with STZ. Compared with negative control rats (NC group), STZ injection led to a dramatically higher intake of daily food and water but a stable blood glucose (Additional file 1: Fig. S1D–F). Twelve weeks later, rats receiving STZ showed decreased thermal sensitivity and motor ability but increased mechanical sensitivity (Fig. 1G–I) which suggested the development of DPN. Hplasma-exos obtained from healthy rats were subjected to Transmission Electron Microscope (TEM) and NTA analysis. The results showed a high concentration of exosomes, and the particle size was approximately 112.27 ± 12.4 nm. The expression of the classic exosome proteins CD9, CD63 and TSG101 was verified by Western blotting (Fig. 1A–C). Next, miRNA sequencing was performed with plasma exosomes from DPN rats and age-matched healthy rats, and significant changes in miRNA expression levels were observed (Fig. 1D, E). Specifically, compared with hplasma-exos, there were 42 upregulated and 33 downregulated miRNAs in DPN plasma-exos.

Then, to investigate whether hplasma-exos play a role in alleviating DPN, 300 µl hplasma-exos (100 µg) or saline was intravenously injected into DPN or NC rats twice a week starting from the 13th week after STZ injection by tail vein (Fig. 1F). Compared with those in the DPN + Saline group, the mechanical sensitivity and thermal sensitivity thresholds in the DPN + Exo group were closer to those in the NC group, and the running time on the rotarod was significantly prolonged (Fig. 1G–I). MCV and SCV were measured before and after exosomes injection and were significantly increased in the DPN + Exo group (Fig. 1J–K). To verify that these behavioral changes were caused by hplasma-exos, exosomes were labeled with EvLINK555. Immunostaining showed that labeled exosomes were internalized by sciatic nerves labeled with neurofilament-L (NF-L) both in DPN rats (Fig. 1L) and in NC rats (Additional file 1: Fig. S2A), indicating that the internalization of exosomes exists under both physiological and pathological conditions. However, hplasma-exos treatment did not reduce random blood glucose and total cholesterol levels in DPN rats (Additional file 1: Table S1). In summary, these results suggest that hplasma-exos could be internalized by the sciatic nerve to improve nerve function in DPN rats.

### Plasma exosomes reduce peripheral nerve injury in DPN

**Fig. 2:**
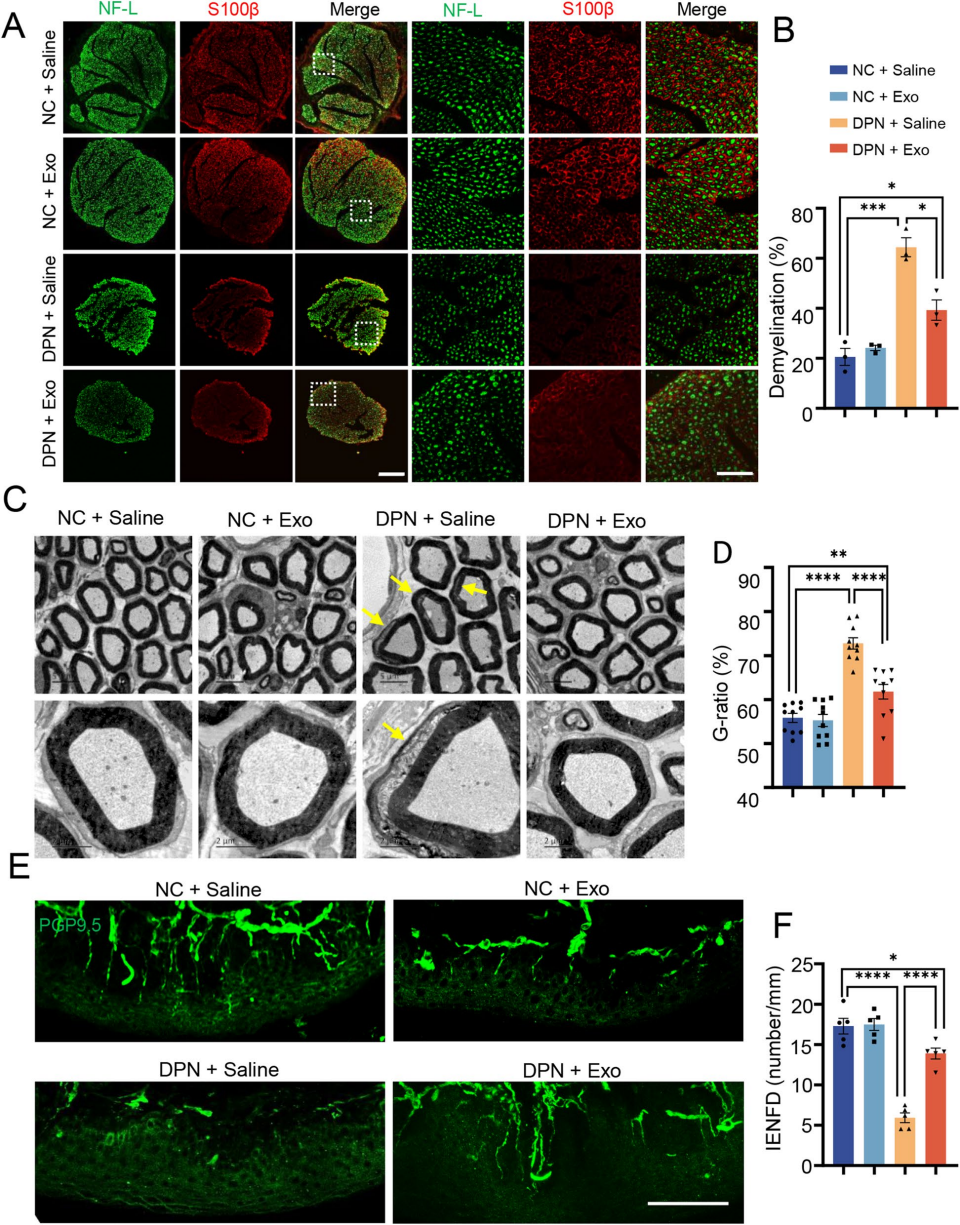
Healthy plasma-derived exosomes reduce sciatic nerve injury in DPN. **A** Representative immunofluorescent staining images of sciatic nerve axons (green) and myelin (red) in different groups, scale bar = 200/50 µm. Squares, images enlarged in right panel. **B** Statistical histogram of the proportion of demyelinating axons in different groups, n = 3. **C** The ultrastructure of sciatic nerves was observed by TEM in different groups of rats, scale bar = 5/2 µm. The yellow arrows point to the site of abnormal demyelination. **D** Quantitative of G-ratio in different groups, n = 10. **E** Representative images showing PGP9.5 positive intraepidermal nerve fibers in the posterior plantar skin of different groups of rats, scale bar = 100 μm. **F** The IENFD analysis is shown in **F**, n = 5. Data are presented as the mean ± SEM. (*p < 0.05, **p < 0.01, ***p < 0.001, ****p < 0.0001)

Since the symptoms of DPN were alleviated by hplasma-exos, we performed histological analysis of sciatic nerves. The transections of sciatic nerves from different groups were immunofluorescence stained with NF-L and S100β (a marker of Schwann cells). The results showed that sciatic nerves in the DPN groups were smaller in size than those in the NC groups. Myelination identified with S100β+ staining around the nerve fibers was impaired in the DPN groups, but improved by treatment with hplasma-exos, with a reduced demyelination percentage compared to that in the DPN + Saline group (Fig. 2A, B). To further examine myelination, the ultrastructure of sciatic nerves was observed by TEM, which showed myelination degeneration in the DPN + Saline group. Again, after hplasma-exos treatment, demyelination was partially recovered, and the g-ratio decreased to 61.81 ± 1.67% (Fig. 2C, D).

The intraepidermal nerve fiber density (IENFD), which innervates the dermis and epidermis, has been used in the diagnosis of peripheral neuropathy. Therefore, we performed immunostaining of foot pad tissues from different groups with PGP9.5. Compared with the NC group, rats in the DPN + Saline group showed a significant decrease in PGP9.5-positive IENFD. However, this reduction was largely rescued by the administration of hplasma-exos, with a density of 13.89 ± 0.67 per millimeter in the DPN + Exo group (Fig. 2E, F).

### Plasma exosomes improve blood flow on the sciatic nerve surface and plantar skin of DPN rats

**Fig. 3:**
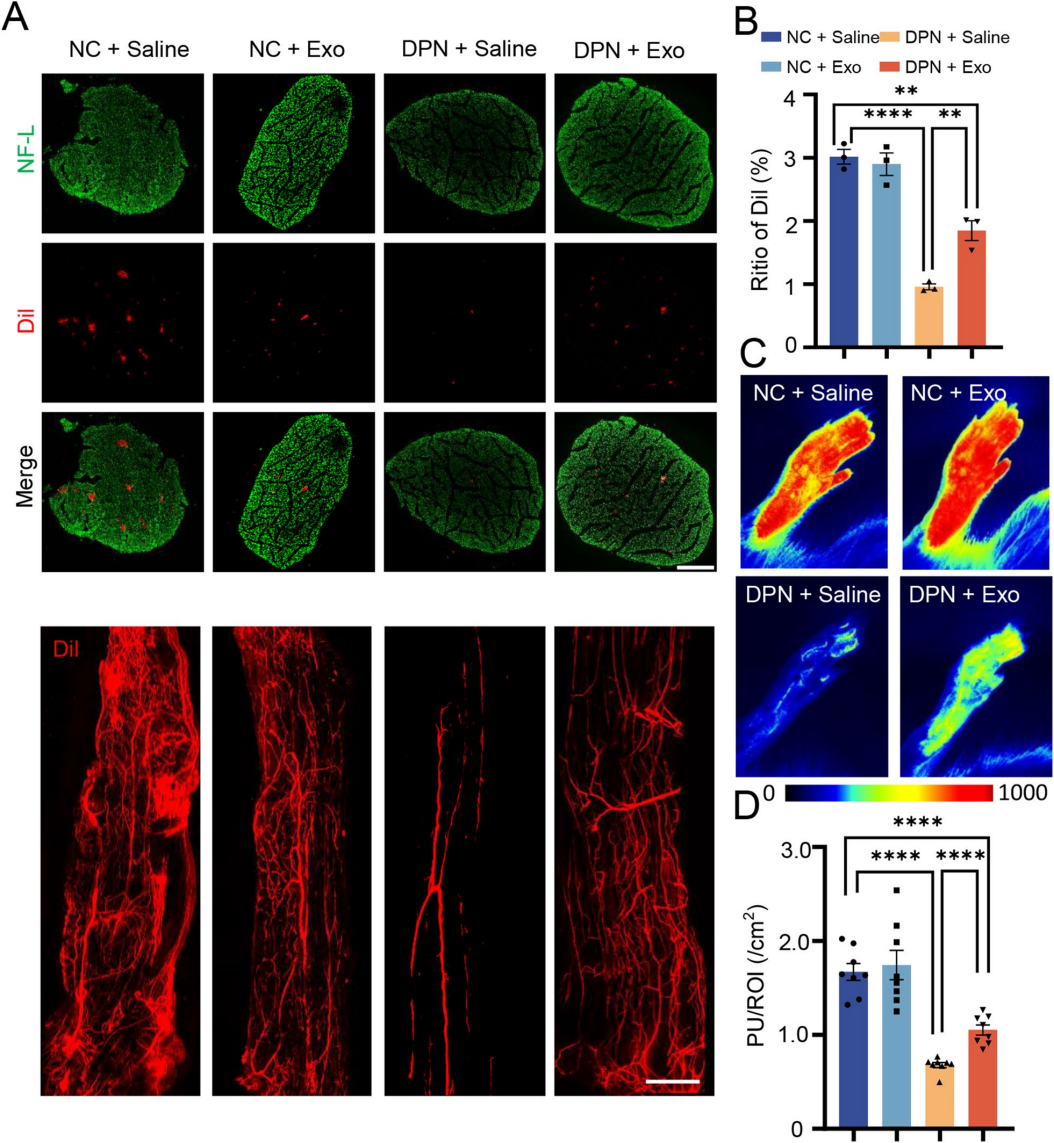
Plasma-exos improves blood flow on the foot and sciatic nerve surface of DPN rats. **A** Representative images of blood vessels of sciatic nerve labeled by Dil (red) and Anti-NF-L (green). Transverse section, scale bar = 200 μm and longitudinal section, scale bar = 1000 μm. **B** Statistical analysis of Dil (red) fluorescence area ratio in transverse section, n = 3. **C** Representative images of plantar laser blood imaging in different groups of rats. **D** The statistical analysis of plantar blood perfusion per unit area is shown in **D**, n = 8. Data are presented as the mean ± SEM. (*p < 0.05, **p < 0.01, ***p < 0.001, ****p < 0.0001)

 Next, we explored the blood flow of the sciatic nerve and plantar skin in DPN rats, as reduced distal blood supply was the most common cause of DPN. Through labeling and visualization of blood vessels with DiI, it was found that blood vessels in the DPN + Saline group decreased significantly regardless of transverse section or longitudinal view in sciatic nerves. However, this decrease was improved by hplasma-exos treatment (Fig. 3A, B). The local blood perfusion of plantar skin was detected by a laser speckle flow imaging system, and the blood perfusion units significantly decreased to 0.68 ± 0.03/cm^2^ in DPN rats. However, this value increased to 1.05 ± 0.05 /cm^2^ after hplasma-exos injection (Fig. 3C, D). Together, these results suggested that hplasma-exos improved distal blood flow in DPN rats.

### Plasma exosomes augment the motor and sensory innervation of the targets

**Fig. 4:**
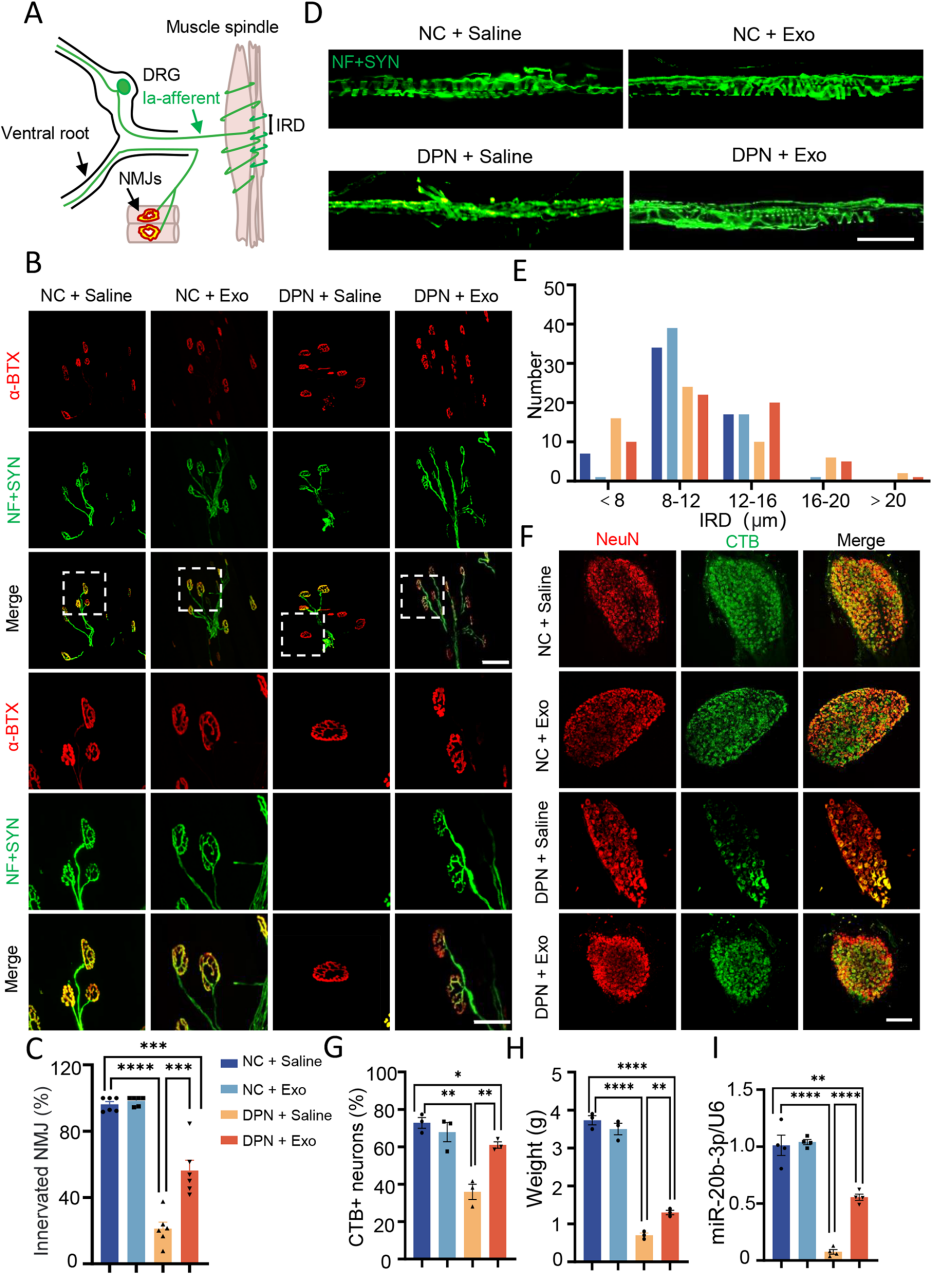
Plasma-exos augments the motor and sensory innervation of the targets. **A** A schematic of NMJ and muscle spindle. **B** Representative images of NMJ, labeled with 555-a-BTX (red) and NF-L + Syn (green) in different groups, bar = 100/50 µm. Squares, images enlarged as shown below. **C** Statistical analysis of the proportion of innervated NMJ (partial denervation and total denervation are excluded), n = 6. **D** Representative images of annulospiral endings of muscle spindles in different groups, bar = 200 μm. **E** Number of muscle spindles in different IRD distribution intervals. **F** Representative images of different groups of neurons labeled with NeuN (red) and retrograde tracer FITC-CTB (green), bar = 400 μm. **G** Statistical analysis of the proportion of FITC-CTB positive cells, n = 3. **H** Statistical analysis of wet weight of gastrocnemius muscle in each group, n = 3. **I** Changes of miR-20b-3p expression in sciatic nerve of rats in each group (**G**), n = 4. Data are presented as the mean ± SEM. (*p < 0.05, **p < 0.01, ***p < 0.001, ****p < 0.0001)

 Muscles are innervated by motor neurons for contraction and sensory neurons for proprioception. The neuromuscular junction (NMJ) formed between motor nerve terminals and muscle fibers is damaged early in the development of the disease, possibly because it is located at the very end of motor nerve conduction [4] (Fig. 4A). By immunofluorescence staining with NF-L + Synapsin-1 (Syn) to label presynaptic terminals and 555-a-BTX (a-BTX) label acetylcholine receptors (AChRs), we found a variety of denervation and fragmentation of NMJs in the DPN + Saline group (Fig. 4B), with a decreased innervation rate of 21.18 ± 4.0%. After hplasma-exos treatment, the proportion of innervation increased to 56.19 ± 6.3% (Fig. 4C).

Unlike changes in the small nerve fibers that govern the perception of pain and temperature, changes in the larger fibers lead to proprioceptive deficits, resulting in the damage of sensory innervation to the muscle spindle [19] (Fig. 4A). By whole-mount staining of extensor digitorum longus (EDL) muscles with anti-NF-L and anti-Syn, muscle spindles could be histologically identified with annulospiral endings at the equatorial region. Accordingly, muscle spindles in the NC groups had characteristic annulospiral endings, whereas these regular coiling structures were disrupted in the DPN rats. However, the treatment with hplasma-exos protected the annulospiral endings of muscle spindles from degeneration (Fig. 4D). We also quantified the distance between the Ia axonal annulospiral rotations (IRD) of each muscle spindle [20]. In contrast to the NC group, whose IRDs were mainly concentrated in the 8–16 μm range, there was a high proportion of IRDs larger than 16 μm or smaller than 8 μm in the DPN + Saline group. As expected, this abnormal innervation, to some extent, was improved after hplasma-exos treatment (Fig. 4E).

To further study the innervation of target muscles, 10 µl of 0.4 mg/ml FITC-cholera toxin subunit B (CTB) was injected into the tibialis anterior (TA) muscles to allow retrograde tracing. One week later, the L3 dorsal root ganglions (DRG) were harvested and immunostained with anti-NeuN, and the percentage of FITC-CTB-labeled neurons was quantified. The results showed that, compared with the NC groups with almost 80% of neurons labeled by CTB, there were only 35.94 ± 4.06% of CTB-positive cells in DPN + Saline rats. However, this number was significantly increased to 60.97 ± 1.7% in the DPN rats receiving hplasma-exos treatment (Fig. 4F, G). Consistently, the wet weight of the gastrocnemius was 3.7 ± 0.12 g in the NC group and 0.7 ± 0.06 g in the DPN + Saline group, whereas it increased to 1.3 ± 0.06 g in the DPN + Exo group (Fig. 4H). Together, these results suggested that hplasma-exos could alleviate the denervation of target muscles in DPN rats. Lastly, the biological distribution of hplasma-exos in DPN and NC rats was investigated using an in vivo imaging system (IVIS) and by staining sections of different tissues. Exosomes were mainly accumulated in the liver and spleen, and a small amount concentrated in the lung and sciatic nerve of both the NC group and the DPN group. These exosomes exhibited a minimum metabolic time of 48 h (Additional file 1: Fig. S3A–C).

### Treatment with miR-20b-3p agomir improves peripheral neuropathy in diabetic rats

**Fig. 5:**
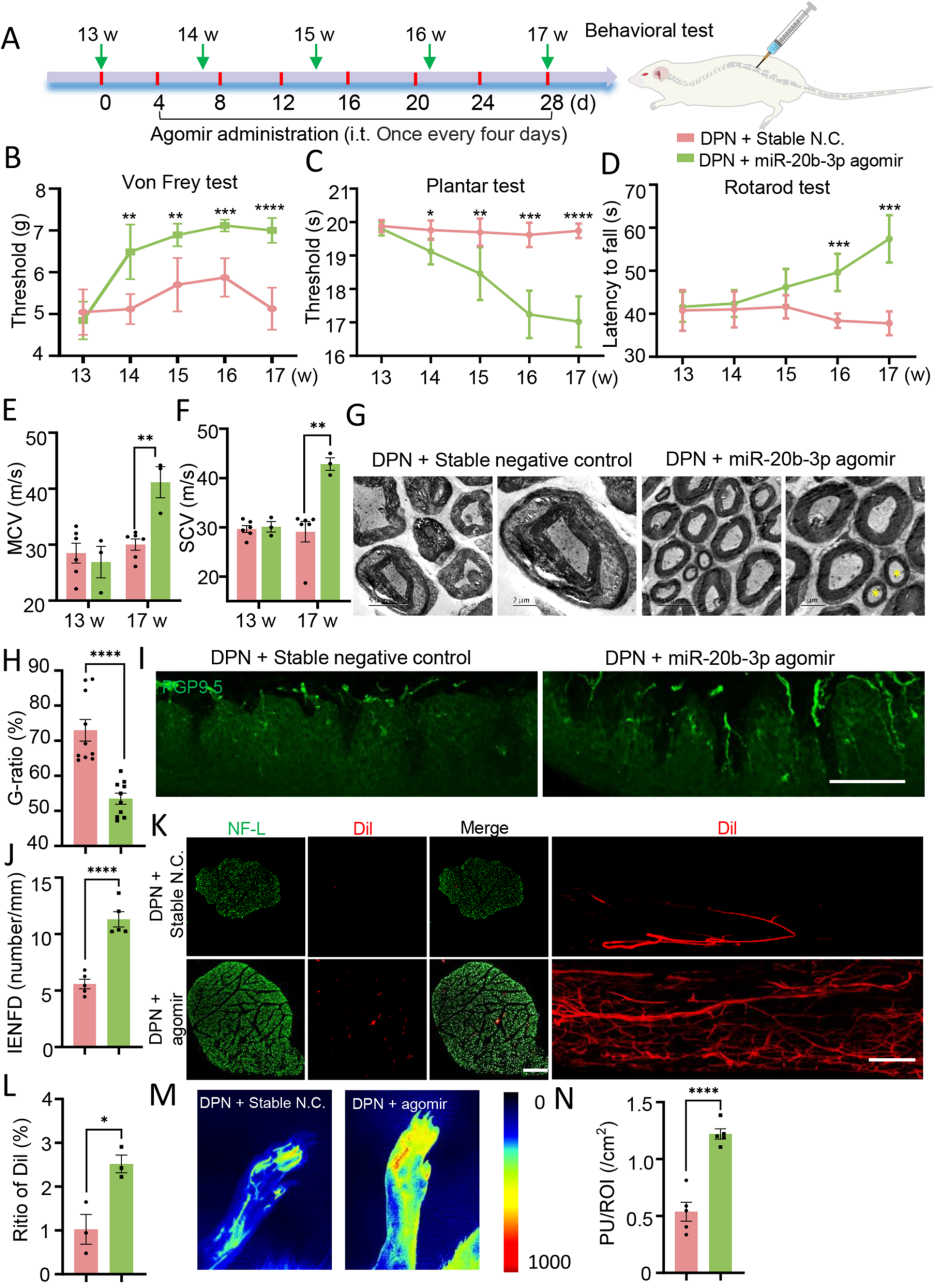
MiR-20b-3p agomir improve nerve damage induced by high glucose. **A** A schematic showing the process of miR-20b-3p agomir administration. **B**–**D** Changes in Von Frey test, plantar test and rotarod test after the treatment of DPN rats with miR-20b-3p stable N.C. or miR-20b-3p agomir, n = 5. **E**, **F** Changes of MCV and SCV after miR-20b-3p agomir treatment, n = 3, 6. **G** The ultrastructure of sciatic nerves was observed by TEM in different groups of rats, scale bar = 5 μm/2µm. The new myelin sheath is marked with *. **H** Histogram represents the quantitative data of the G-ratio under various conditions, n = 10. **I** Representative images showing PGP9.5 positive intraepidermal nerve fibers in the posterior plantar skin of different groups of rats, scale bar = 100 μm. **J** Statistical analysis of IENFD is shown in **J**, n = 5. **K** Representative images of Dil perfusion in different groups of transverse and longitudinal section. Transverse section, scale bar = 200 μm and longitudinal section, scale bar = 1000 μm. **L** Statistical analysis of Dil (red) fluorescence area ratio in transverse section, n = 3. **M** Representative images of plantar blood flow imaging in different groups of rats. **N** Statistical analysis of plantar blood perfusion per unit area is shown in **N**, n = 5. **B**–**D** Data are presented as the mean ± SD, others are presented as the mean ± SEM. (*p < 0.05, **p < 0.01, ***p < 0.001, ****p < 0.0001)

 MiRNAs are exosome cargos that can alter the gene expression of recipient cells and influence cellular processes, therefore, we asked whether hplasma-exos function through miRNAs. As miR-20b-3p was the most differentially expressed miRNA between the two groups (Fig. 1D), we first checked the expression of miR-20b-3p in each group. The sciatic nerves were harvested and subjected to qRT-PCR, miR-20b-3p in the NC + Saline group was normalized to 1 and was significantly decreased in the DPN + Saline group. However, the administration of hplasma-exos reversed the reduced expression of miR-20b-3p in DPN rats (Fig. 4I). Therefore, we studied the role of exogenous miR-20b-3p in DPN. DPN rats at 13 weeks after STZ administration began to receive intrathecal injection of 2 nM miR-20b-3p agomir or stable negative control (stable N.C.) every four days, with a treatment cycle of 28 days (Fig. 5A). Behavior tests were carried out from the 13th week and continued every week during the treatment. Compared with stable N.C., abnormal mechanical and thermal sensitivity tended to normalize, and reduced motor ability was improved in the miR-20b-3p agomir group (Fig. 5B–D). Consistently, the MCV and SCV were also increased after miR-20b-3p agomir treatment (Fig. 5E, F). New myelination occurred after treatment with miR-20b-3p agomir by TEM (Fig. 5G), with the g-ratio decreasing to 53.47 ± 1.59% (Fig. 5H). In addition, the IENFD in the foot pads and the blood perfusion in the sciatic nerve and plantar skin were all increased in the agomir-treated DPN rats (Fig. 5I–N).

**Fig. 6:**
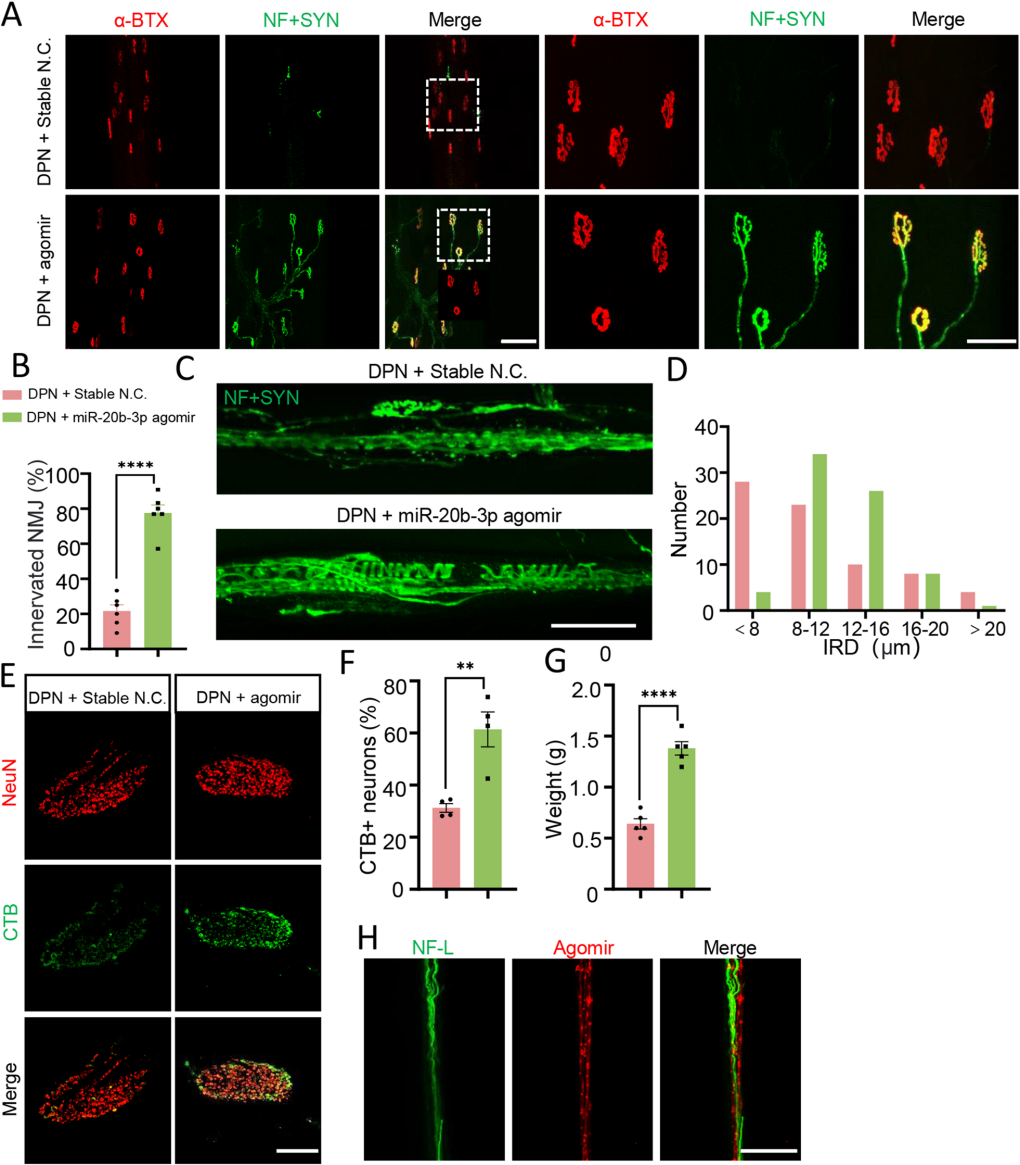
MiR-20b-3p agomir augments the motor and sensory innervation of the targets. **A** Representative images of NMJs labeled with 555-a-BTX (red) and NF-L + Syn (green) in different groups, scale bar = 100/50 µm. Squares, images enlarged in right panel. **B** Statistical analysis of the proportion of innervated NMJs (partial denervation and total denervation are excluded), n = 6. **C** Representative images of annulospiral endings of muscle spindles in different groups, scale bar = 200 μm. **D** Number of muscle spindles in different IRD distribution intervals. **E** Representative images of different groups of neurons labeled with NeuN (red) and retrograde tracer by CTB (green), scale bar = 500 μm. **F** Statistical analysis of the proportion of CTB positive cells, n = 4. **G** Statistical analysis of wet weight of gastrocnemius muscle in each group, n = 5. **H** Red fluorescently labeled agomir was found in the NF-L-labeled sciatic nerve. Data are presented as the mean ± SEM. (*p < 0.05, **p < 0.01, ***p < 0.001, ****p < 0.0001)

 We also found that the proportion of innervated NMJs increased after treatment with miR-20b-3p agomir (Fig. 6A, B). Regular annulospiral endings were observed in the miR-20b-3p agomir-treated rats, with IRDs mainly concentrated within 8–16 μm (Fig. 6C, D). After injecting FITC-CTB for retrograde tracing, more CTB + DRG neurons in the miR-20b-3p agomir group were observed (Fig. 6E, F). Additionally, the wet weight of the gastrocnemius muscle also increased (Fig. 6G). However, compared with the control group, miR-20b-3p agomir treatment did not significantly change the levels of blood glucose or total cholesterol at the end of the experiment, indicating that exogenous miR-20b-3p did not play a therapeutic role by reducing the levels of glucose and other related metabolites (Additional file 1: Table S2). To ensure that these changes were caused by agomir, sciatic nerves were stained and red fluorescently labeled miR-20b-3p agomir was mainly in or around the sciatic nerve (Fig. 6H). Together, these results suggested that miR-20b-3p improved peripheral neuropathy and tissue damage in diabetic rats.

### MiR-20b-3p downregulates the expression of Stat3 and promotes autophagy in Schwann cells

**Fig. 7:**
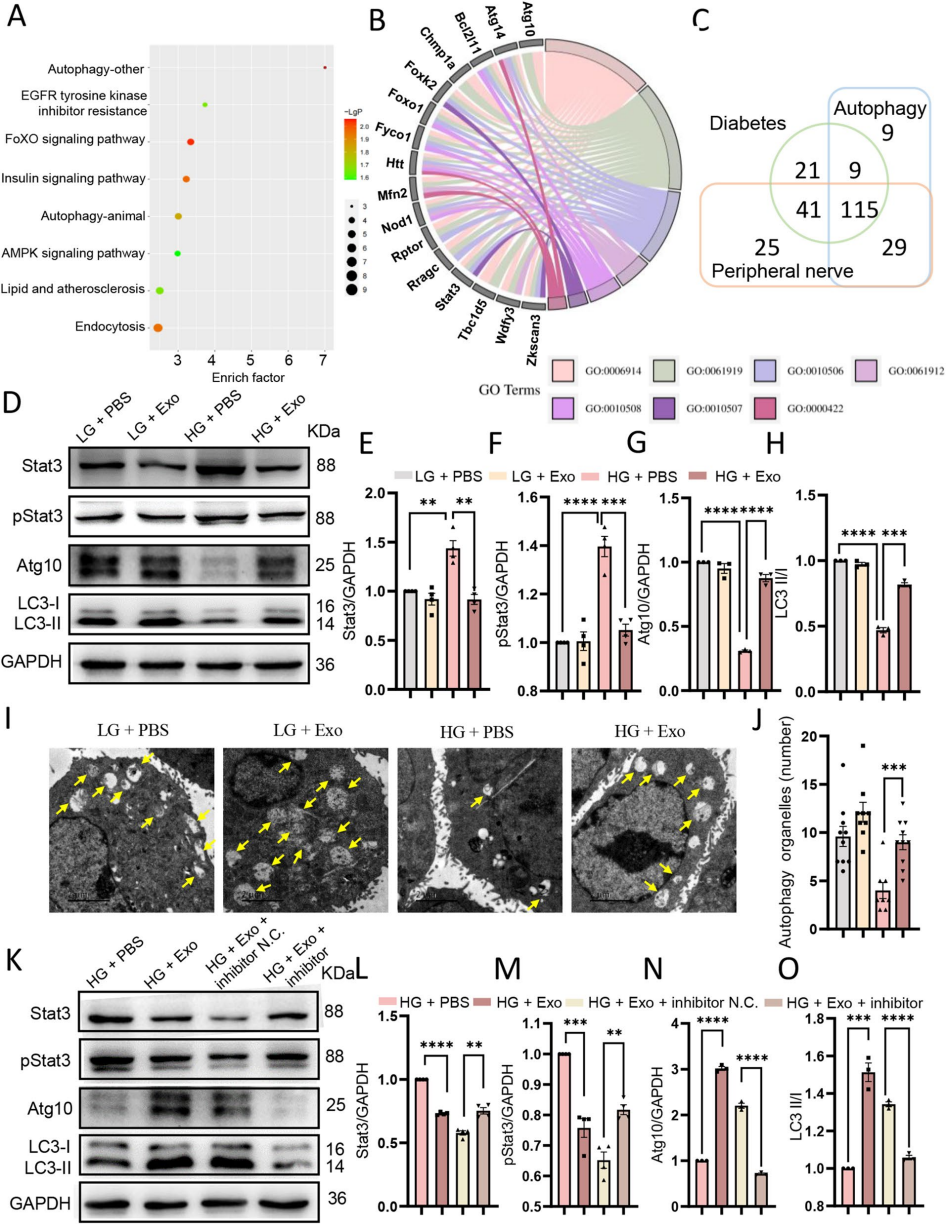
Plasma-exos improves RSC96 autophagy under high glucose stimulation. **A** Bubble map for KEGG analysis of miR-20b-3p. **B** Chordal graph of GO enrichment analysis of target genes of miR-20b-3p in autophagy related pathways. (GO:0006914 autophagy, GO:0061919 process utilizing autophagic mechanism, GO:0010506 regulation of autophagy, GO:0061912 selective autophagy, GO:0010508 positive regulation of autophagy, GO:0010507 negative regulation of autophagy, GO:0000422 autophagy of mitochondrion). **C** Venn diagram of the 115 matched targets between the diabetes, autopaghy and peripheral nerve targets. **D** Grey strips by western blot of the expression of Stat3, pStat3, Atg10, LC3 II/I at different groups in RSC96 in vitro after stimulation with hplasma-exos. **E**–**H** Quantification of the related expression of Stat3 (**E**), pStat3 (**F**), Atg10 (**G**) and LC3 II/I (**H**) in RSC96 after stimulation with PBS or hplasma-exos, n = 3. **I** TEM images of autophagy organelles in RSC96 cells with different stimuli, bar = 2 μm. Yellow arrows, autophagy organelles. **J** Statistical analysis of the number of autophagy organelles in indicated groups, n = 8–10. **K** Grey strips by western blot of the expression of Stat3, pStat3, Atg10, LC3 II/I at different groups in RSC96 in vitro. **L**–**O** Statistical results of the expression of Stat3 (**L**), pStat3 (**M**), Atg10 (**N**) and LC3 II/I (**O**) at different groups in RSC96 after different stimuli, n = 3–4. *HG* high glucose environment, *LG* low glucose environment. Data are presented as the mean ± SEM. (*p < 0.05, **p < 0.01, ***p < 0.001, ****p < 0.0001)

 Next, we investigated the underlying mechanism of miR-20b-3p in alleviating DPN. Through KEGG and GO analysis, miR-20b-3p was found to be correlated with autophagy, a process that removes myelin debris from Schwann cells and consumes the inflammasome after sciatic nerve injury (Fig. 7A, B). Stat3 was correlated with multiple pathways, including autophagy, through gene enrichment analysis, and Stat3 was the most correlated gene among the genes associated with negative regulation of autophagy predicted by Targetscan software (Fig. 7B). In Wayne’s analysis, we selected target genes related to peripheral nerves, autophagy and diabetes, including Stat3 (Fig. 7C). Therefore, we explored whether miR-20b-3p and Stat3 could affect the autophagy of Schwann cells.

We first investigated whether hplasma-exos correlated with autophagy. To mimic the diabetic microenvironment, RSC96 cells were cultured with 100 mM glucose alone or together with 20 µg/ml hplasma-exos. Autophagy was indicated by the ratio of LC3 II to LC3 I, as well as the expression of Atg 10. Western blot analysis showed that LC3 II/I and Atg10 were reduced by high glucose, suggesting reduced autophagy. However, Stat3, a negative regulator of autophagy, was increased. Intriguingly, after hplasma-exos treatment, the enhanced Stat3 and phosphorylation of Stat3 decreased, while LC3 II/I and Atg10 became increased (Fig. 7D–H). Additionally, the number of autophagy organelles was quantified by TEM. The results showed that more autophagy organelles were observed after hplasma-exos treatment (Fig. 7I, J). We also performed TUNEL staining of RSC96 cells in different groups and found that exosomes treatment reduced the apoptosis of Schwann cells induced by high glucose (Additional file 1: Fig. S4A, B). By in vitro culture of DRG neurons from different groups, the axon outgrowth labeled by anti-NF-L was inhibited by high glucose. However, treatment with plasma-exos led to a slight recovery of axon outgrowth (Additional file 1: Fig. S4C, D).

Then, a miR-20b-3p inhibitor was employed to interfere with its expression in hplasma-exos. Quantified RT-PCR showed that miR-20b-3p was significantly decreased after transfection of the inhibitor (Additional file 1: Fig. S5A), and the increased autophagy was disrupted as indicated by reduced Atg 10 and LC3 II/I and increased Stat3 and phosphorylation of Stat3 (Fig. 7K–O).

**Fig. 8:**
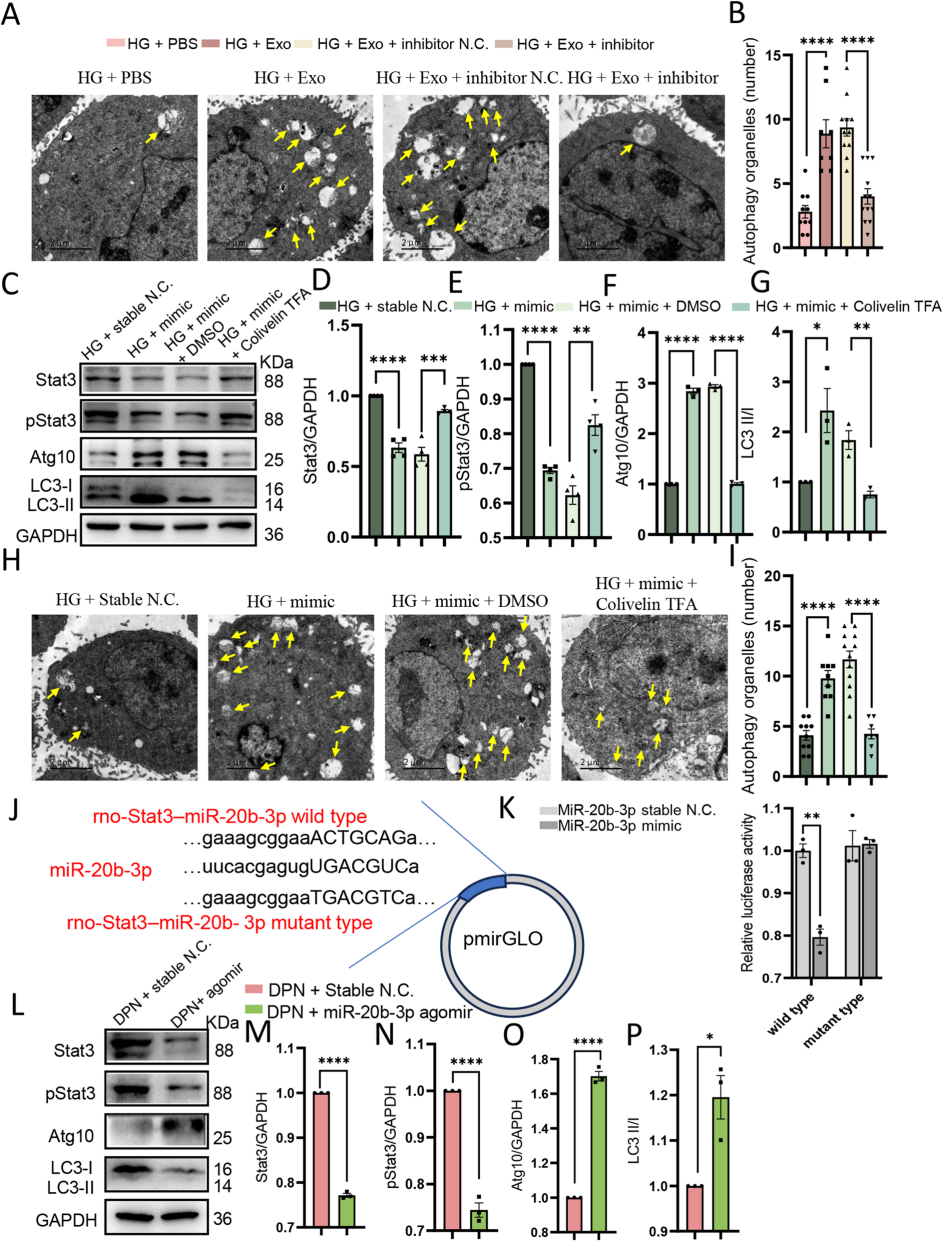
MiR-20b-3p down-regulates the expression of Stat3 and promotes autophagy in Schwann cells. **A** TEM images of autophagy organelles in RSC96 cells with different stimuli, bar = 2 μm. Yellow arrows, autophagy organelles. **B** Statistical analysis of the number of autophagy organelles in indicated groups, n = 8–12. **C** Grey strips by western blot of the expression of Stat3, pStat3, Atg10, LC3 II/I at different groups in RSC96 in vitro. **D**–**G** Statistical results of the expression of Stat3 (**D**), pStat3 (**E**), Atg10 (**F**) and LC3 II/I (**G**) at different groups in RSC96 after different stimuli, n = 3. **H** TEM images of autophagy organelles in RSC96 cells with different stimuli, bar = 2 μm. Yellow arrows, autophagy organelles. **I** Statistical analysis of the number of autophagy organelles in indicated groups, n = 8–12. **J** Complementary sequences between miR-20b-3p and the 3′-untranslated region (UTR) of wild type or mutant type of Stat3 were obtained. **K** Target relationship between miR-20b-3p and Stat3 was assessed by dual luciferase reporter gene assay. **L**–**P** Quantitative analysis and statistical results of the expression of Stat3, pStat3, Atg10, LC3 II/I at different groups in vivo by Western blot, n = 3. *HG* high glucose environment, *LG* low glucose environment. Data are presented as the mean ± SEM. (*p < 0.05, **p < 0.01, ***p < 0.001, ****p < 0.0001)

Again, we also verified the change of autophagy by quantifying the number of autophagy organelles using TEM (Fig. 8A, B). To further check whether Stat3 was required for the regulation of autophagy by miR-20b-3p, Colivelin TFA (50 µg/mL), an agonist of Stat3, was used alone or together with miR-20b-3p mimic, The expression of miR-20b-3p was significantly increased after transfection with miR-20b-3p mimic (Additional file 1: Fig. S5B). In accordance with plasma-exos, the mimic resulted in enhanced autophagy and decreased Stat3. However, after the addition of Colivelin TFA to activate Stat3, the promotion effect on autophagy was aborted, as indicated by the reduced Atg10 and LC3 II/I, as well as autophagy organelles. (Fig. 8C–I). Subsequent dual luciferase reporter gene analysis revealed that Stat3 was indeed a direct target of miR-20b-3p (Fig. 8J, K).

Furthermore, we analyzed the expression of related proteins in vivo and found that Stat3 in the sciatic nerve of DPN rats was high and the autophagy level was low, whereas this situation was reversed after treatment with miR-20b-3p agomir (Fig. 8L–P). We also conducted an analysis of the pStat3/Stat3 values across various groups. The results revealed no substantial variation among the groups, suggesting that miR-20b-3p primarily influences the expression of Stat3 without significantly affecting its phosphorylation level (Additional file 1: Fig. S6A–D). In total, these results suggested that miR-20b-3p downregulated the expression of Stat3 and thus promoted autophagy in Schwann cells, which ultimately improved DPN-related neurological symptoms.

### Ageing plasma exosomes had no positive effect on the improvement of DPN

Since the plasma-exos were from healthy rats, we next asked whether ageing plasma-exos also had a similar function. Ageing plasma-exos were isolated from 24-month-old rats and intravenously injected into DPN rats twice a week from the 13th week after STZ treatment. Plasma-exos obtained from ageing rats were subjected to TEM and NTA analysis. The results show that the particle size of the extracted exosome is distributed within 30–200 nm. The expression of the classic exosome proteins CD9, CD63 and TSG101 was verified by Western blotting (Additional file 1: Fig. S7A–C). Ageing plasma-exos had no effect on peripheral neurological function as assayed by the von Frey test, plantar, rotarod test, MCV and SCV (Additional file 1: Fig. S8A–C). TEM showed that the myelin lamellar structure of DPN rats was loose, myelin shape was irregular, and mitochondrial vacuolation worsened after exogenous administration of ageing plasma-exos, but there was no significant difference in the g-ratio between the two groups (Additional file 1: Fig. S8D, E). Additionally, there was no difference in IENFD between these two groups (Additional file 1: Fig. S8F, G). Compared with the saline group, the proportion of DiI perfused blood area in sciatic nerves was slightly decreased in the ageing plasma-exos treatment group, while there was no difference in plantar skin (Additional file 1: Fig. S8H–J). NMJs staining showed that most NMJs were denervated in DPN rats, and the innervation slightly decreased in the ageing plasma-exos group (Additional file 1: Fig. S9A, B). For the muscle spindle, the distance between adjacent annulospiral endings was either too large or too small in both groups (Additional file 1: Fig. S9C, D). Consistently, there was no difference in the proportion of DRG neurons retrogradely labeled by FITC-CTB (Additional file 1: Fig. S9E, F) or the wet weight of the gastrocnemius between the two groups (Additional file 1: Fig. S9G). Finally, qRT-PCR was carried out to detect miR-20b-3p in vitro after the addition of ageing plasma-exos, and no difference was observed between these two groups, which might be the reason why ageing plasma-exos did not improve DPN symptoms (Additional file 1: Fig. S9H). Of note, deaths of unknown causes also occurred in the ageing plasma-exos treatment group. Together, these results suggested that ageing plasma-exos could not benefit DPN and in some ways might promote the progression of DPN.

## Discussion

This study found that miRNAs in DPN plasma-exos are quite different from those of healthy plasma-exos in rats. DPN rats receiving hplasma-exos showed an improvement in peripheral neuropathy, with enhanced myelination, IENFD, blood perfusion and motor or sensory neuron innervation. MiR-20b-3p was one of the top enriched miRNAs in hplasma-exos and showed a similar performance in alleviating neuropathy symptoms to hplasma-exos when given to DPN rats. Mechanistic studies found that Stat3 was a target gene of miR-20b-3p and negatively related to autophagy. By reducing the expression of Stat3, miR-20b-3p promoted high glucose-induced cell autophagy, which might contribute to DPN recovery. These new findings elucidated a novel strategy for DPN treatment.

Blood therapy is a promising treatment, and multiple studies have found that heterochronic parabiosis can restore a youthful state to older mice [12, 14, 15]. Platelet-rich plasma also has very important applications in peripheral nerve fields [21, 22]. Some very recent studies have found that platelet factor 4 (PF4) in young blood improves motor and cognitive performance. Villeda reported that the plasma composition in young individuals was different from that of older ones, and found that exogenous PF4 improved neuroinflammation and increased synaptic plasticity [23]. Klotho is a “longevity factor” that has long attracted attention, and Professor Dubal reported that its role in promoting neural connections is revealed by activating PF4 [24]. Tara Walker considered that platelets release PF4 into the bloodstream after exercise to enhance cognitive function [25]. All of which goes to show that there’s plenty of potential for exploration in the blood. Here, we extracted plasma exosomes from juvenile rats and employed them to treat DPN rats. Consistent with the above studies, we found an improvement of neuropathy in DPN rats, which, however, was not observed when exosomes were isolated from ageing rats.

Exosomes, as a promising treatment strategy for multiple diseases, have played an important role in the field of peripheral nerves [26–28], including the treatment of DPN [9, 10]. MicroRNAs contained within exosomes have been reported to promote the recovery of neural function [29, 30]. Pusic suggests that serum exosomes from young animals and their delivery of miR-219 may be a useful treatment for remyelination [31]. Nerve damage induced by high glucose often leads to myelin structural disorders [32, 33]. In this study, we found that healthy plasma-exos could significantly improve peripheral neuropathy in diabetic rats by carrying miR-20b-3p, including the promotion of remyelination. A decrease in IENFD can lead to dysfunction of small nerve fibers [34], whereas lesions to larger nerve fibers block conduction velocity [35]. In DPN rats, microvessels also undergo a series of changes, accelerating the progression of DPN [9, 36], thus, the restoration of blood perfusion plays an important role in the treatment of diabetes. Clinical observation shows that the sensory system is damaged before the motor neurons. Due to the interaction between the peripheral nervous system (PNS) and the central nervous system (CNS) for the control of movement, this motor deficit can occur regardless of whether the PNS and CNS are affected [4]. Therefore, apart from MCV, SCV, IENFD, and blood perfusion, the therapeutic effects of exosomes on sensory or motor innervation have also been explored.

In this study, we observed that both the NC and DPN groups exhibited internalization of hplasma-exos by the sciatic nerve. However, the administration of exosomes did not induce any changes in the related proteins of the internal environment in the NC group. This lack of response may be attributed to the presence of homeostasis in the normal tissue microenvironment, which remains unaffected by the addition of exogenous substances. Conversely, in pathological tissues, the microenvironment’s homeostasis is disrupted, and the administration of exogenous repair substances can help restore it. Nevertheless, identifying methods to enhance the targeting of exosomes to diseased tissues remains a challenge that we will address in subsequent research endeavors.

MiRNAs are noncoding endogenous RNAs that regulate gene expression after transcription [37]. MiR-20b-3p is a rarely reported miRNA that is significantly decreased in diabetic rat tissues [38, 39]. Consistent with this finding, we identified miR-20b-3p as the most reduced miRNA in DPN plasma-exos. In human glioblastoma, Lnc-TALC promotes c-Met expression and thus Stat3 phosphorylation by competitively binding to miR-20b-3p, which is associated with temozolomide resistance [40]. However, in rats, we found that stat3 was the target gene of mir-20b-3p through prediction and dual luciferase reporter analysis and was negatively regulated by mir-20b-3p. A plausible explanation for this might be that the sequence of miR-20b-3p varies among species.

Stat3, as a transcription regulator, plays an essential role in many biological processes involving axon regeneration, microvascular endothelial cell migration, and tube formation [41, 42]. Autophagy of Schwann cells is essential for the degradation and removal of myelin fragments after peripheral nerve injury [43]. In DPN, Schwann cell autophagy decreases significantly, and promoting Schwann cells autophagy in various ways can significantly improve DPN [44–46]. Inhibition of p-Stat3 was reported to improve autophagy inhibition in Schwann cells in a high glucose state [47]. Consistent with this, our study suggested that miR-20b-3p could downregulate the expression of Stat3 and consequently reduce pStat3, thereby promoting autophagy and improving the neurological symptoms of DPN. However, how to effectively target exosomes to diseased tissues and the mechanism of increasing blood perfusion after plasma-exos was not clearly elaborated in this article, which will also be the focus of our next research. Interestingly, unlike the improvement of DPN by hplasma-exos, we did not observe a remission of DPN symptoms when using ageing plasma-exos, which we propose may be related to miRNA changes in ageing plasma-exos. Since this study is mainly focused on the improvement of DPN in hplasma-exos and explored its mechanism, we did not follow up on the changes in miRNA in ageing plasma-exos. Although the addition of ageing plasma-exos to RSC96 cells in high glucose culture did not change the expression of miR-20b-3p, we could not rule out the possibility that this ineffective effect may be related to changes in other miRNAs.

## Conclusion

In conclusion, our study suggests that delivery of hplasma-exos promotes functional recovery of DPN in diabetic rats. Enrichment of miR-20b-3p in healthy plasma exosomes promotes autophagy of Schwann cells by targeting Stat3 and slows the progression of DPN. Our observations provide a new prospect for the application of plasma exosomes.

## Materials and methods

### Animals

Male Sprague-Dawley (SD) rats aged 5–6 weeks were selected (ChangChun ISI Biotechnology Co., Ltd.) and STZ (Sigma, 50 mg/kg) was injected intraperitoneally into rats after one week of acclimation and 24 h of fasting [48, 49]. Blood glucose (Roche blood glucose meter), food intake and water intake were monitored. All animal procedures were conducted in strict accordance with the US National Institutes of health (NIH) Guide for the Care and Use of Laboratory Animals, as defined by the US National Academy of Sciences and approved by the Jilin University Administration Committee of Experimental Animals.

### Isolation and identification of plasma exosomes

SD rats were anesthetized using isoflurane, after the disappearance of the righting reflex, the abdominal cavity was exposed and the surface tissue was wiped with sterile absorbent cotton, and the abdominal aorta was exposed. Abdominal aorta blood was collected by EDTA vacuum vasculature (BD). The samples were centrifuged at 4 ℃ at 1900×*g* for 10 min and 3000×*g* for 15 min to obtain plasma. Exosomes were extracted from the plasma by ultracentrifugation [50]. The obtained exosomes were injected into the target rats via the tail vein with a volume of 300 µl (100 µg) after the determination of exosomes concentration using BCA protein assay kit. We used EvLINK555 (IlluTingo, EL012100211) to label the exosomes according to the instruction and observe their internalization in the sciatic nerve. We also used DiI (Bioss, D-9101) to detect the aggregation of exosomes in different tissues. Exosome incubation methods are detailed in the instructions.

### Behavioral tests

#### Measurement of mechanical sensitivity

The rats to be measured were placed in a box with a metal mesh and left for 15–30 min until the rats were acclimated to the unfamiliar environment. A series of von Frey filaments were selected. Von Frey filaments were applied to the hairless part of the left palmoplanta of rats (Additional file 1: Fig. S1A) with a duration of action of 6–8 s. Lifting or licking of the foot on the stimulated side by the rats was considered positive. Each rat was tested three times, and the average was the final result. The 50% paw withdrawal threshold approach was adopted [51].

### Measurement of thermal sensitivity

After the rats were acclimated to the environment, they were placed onto a plate with a temperature of 53 ℃, and the latency of the rats’ emergence of licking or foot lifting was recorded (Additional file 1: Fig. S1B) [52]. In order to avoid the feet of the rats being burned, if there was no response within 20 s, the latency was recorded as 20 s [53].

### Rotarod performance

The rats were trained 3 days before measurement. During the test, the rotarod apparatus (Panlab, LE8505) was set to acceleration mode, and the speed increased from 4 r/min to 40 r/min within 5 min (Additional file 1: Fig. S1C). After the rats were acclimated to the environment, the time of the rats on the rotarod was recorded [54].

### Electrophysiological tests

Motor nerve conduction velocity (MCV) and sensory nerve conduction velocity (SCV) were measured by published methods using Portable Medical Electromyographic Evoked Potentiometer (Haishen, NDI-097) [55]. In brief, after anesthesia, the rats were fixed in prone position so that the lower extremity abduction 45° to fully expose the lower extremity muscle group. For motor nerve conduction velocity (MNCV) examination, in order to avoid the influence of neuromuscular junction, the stimulation electrode was placed on the proximal sciatic nerve (groin) and the motor branch of tibial nerve (ankle). The recording electrode was placed on the distal motor branch of the tibial nerve (metacarpophalangeal). Measured the conduction time and distance of the current from the groin to the ankle, and then calculated the nerve conduction velocity. For sensory nerve conduction velocity (SNCV), the stimulation electrode was placed on the distal end of the tibial nerve (metacarpophalangeal), and the recording electrode was placed on the proximal end of the sciatic nerve (groin) to measure the conduction time and distance of the current from the metacarpophalangeal to the groin, and the nerve conduction velocity was measured.

### Transmission electron microscope (TEM)

 In brief, the RSC96 cell and sciatic nerve were fixed with 2.5% glutaraldehyde at 4 ℃ for 24 h. Next, the nerves were washed with phosphoric acid buffer for 10 min, dehydrated in a series of graded alcohol solutions, and finally fixed with epoxy resin embedding agent. After ultrathin sections (50–80 nm) were made, the sections were stained with uranyl acetate-lead citrate, and the alterations of autophagy organelles (isolation membrane, autophagosome and autolysosome) and myelin were observed by TEM (FEI, TECNAI SPIRIT).

### DiI infusion

DiI (Bioss, D-9101) working solution was prepared according to Li [56]. The rats were perfused with 100 ml PBS, 30 ml DiI working solution, and 50–100 ml 4% PFA at a rate of 1–2 ml per minute using a perfusion pump. The sciatic nerves were cut into 10 μm thick transverse sections. Immunofluorescence staining was performed with anti-NF-L, and the proportion of DiI fluorescence area was calculated. Alternatively, the fixed sciatic nerve was directly compressed longitudinally to observe the distribution of blood vessels.

### Plantar blood perfusion volume analysis

The amount of plantar blood perfusion was measured by a laser speckle flow imaging system (RWD). The camera parameters were set (spatiotemporal algorithm, 512 × 512, 120 frames, exposure time 5 ms) and the effective monitoring laser position was adjusted. First, rats were anaesthetized and then draw the ROI region to avoid the interference of hair and other parts, the sensor was placed 10 cm above the region of interest (ROI). The perfusion units (PU) in selected ROIs were recorded.

### Cholera toxin subunit B (CTB) injection and staining

10 µl of 0.4 mg/ml CTB (Absin, Shanghai, China) was injected into the tibialis anterior (TA) muscles [57, 58]. The L3 DRG was separated after one week. Immunofluorescence staining for DRG with anti-NeuN was conducted. The ratio of CTB-positive neurons was quantified by ImageJ.

### In vivo tracing of plasma exosomes

In order to detect the biological distribution of hplasma-exos in vivo, we labeled exosomes using DiR (Absin, abs45153692) and DiI (Bioss, D-9101). The methods of DiR and DiI labeling exosomes are carried out according to an early publication [59]. The biological distribution of DiR-labeled exosomes at different time after injection was measured using an in vivo imaging system (IVIS) (Tanon, ABL X5). Additionally, we observed the distribution of DiI-labeled hplasma-exos in vivo by staining sections of different tissues 6 h after injection.

#### Cell culture

Rat RSC96 Schwann cells and 293T cells were purchased from Procell Life Science & Technology Co., Ltd. Cells were maintained in low (LG, 5.5 mM), high (HG, 100 mM) or normal (25mM) glucose conditions with 10% exosome-free serum. Cells were cultured in a humidified incubator at 37 °C and 5% CO_2_.

DRG neurons were isolated from different groups of rats at the end of the experiment and cultured under different concentrations of glucose according to published methods [60]. Immunofluorescence staining was performed with anti-NF-L after 48 h culture. The longest axon length of DRG neurons was calculated.

### Immunofluorescence staining

The sciatic nerve was separated into a nerve filament or 10 μm thick sections. After blocking by 5% BSA and 1% Triton X-100 for 2 h, primary antibodies against NF-L and S100β in blocking buffer were incubated at 4 °C overnight, and then fluorophore-labeled secondary antibodies (Invitrogen, 1:500) were incubated at room temperature for 90 min. After PBST washing, the samples were mounted with mounting medium and coverslipped. Finally, the sections were photographed by confocal microscopy (Nikon, A1). The ratio of S100β + myelin around the nerve fibers was calculated.

The whole layer of plantar skin was selected with a 3 mm biopsy punch. After 4% PFA fixation and sucrose gradient dehydration, OCT embedding, 50 μm thick sections were obtained by freezing microtome (Leica, CM1950). After blocking, primary antibody PGP9.5 was used for staining, and the remaining procedure was the same as above. The number of nerve fibers crossing the derma-epidermal junction was calculated, and IENFD was expressed as the number of fibers per millimeter [61].

The EDL was torn into a single filament and rinsed with 0.1 M glycine for 30 min before being permeated overnight with 2% Triton X-100. NMJs and muscle spindles were stained with NF-L + Syn to label presynaptic terminals and 555-a-bungarotoxin to label AChRs. The proportion of undenervated NMJs and the shape of muscle spindles were observed, and the IRD was calculated.

#### TUNEL staining

To assess apoptosis, RSC96 cells were stained according to the staining procedure of the TUNEL kit (Beyotime, C1086). The proportion of TUNEL-positive cells was calculated by ImageJ.

#### Luciferase reporter assay

The 3′-UTR wild-type and mutant sequences of Stat3 are detailed in Fig. 8J. 293T cells were inoculated into 24-well plates, transfected with 0.5 µg wild-type (WT) or mutant (MUT) plasmid and 5 pmol miR-20b-3p mimic or stable N.C. using Lipofectamine 3000 (Invitrogen) and incubated for 48 h (change the fluid in 6 h). The double luciferase reporter detection system (Promega, E1910) was used to determine the Firefly and Renilla luciferase activities, and statistical analysis was conducted according to a previously described method [62].

#### MiRNA extraction and RT-PCR analysis

The extraction and reverse transcription of miR-20b-3p in tissues and cells were performed according to the kit instructions (TRANS, ER601, AT351). SYBR GreenER qPCR Mix and specific gene-primers were used for quantitative RT-PCR. Standard PCR was performed at 95 °C for 30 s, 95 °C for 5 s, and 60 °C for 34 s for a total of 40 cycles. The relative expression was calculated by 2^−ΔΔCT^ [63]. Primer sequences are detailed in Additional file 1: Table S3.

### Western blotting

Protein samples were prepared with RIPA lysis buffer (Sangon Biotech, C500008), separated by SDS-PAGE and transferred onto PVDF membranes. After 5% BSA blocking, the membrane was incubated with primary antibodies against CD9 (Santa, SC13118, 1:200), CD63 (Santa, SC5275, 1:200), TSG101 (Santa, SC7964, 1:200), Stat3 (Abmart, T55292, 1:1000), Phosoph-Stat3 (Abmart, T56566, 1:1000), LC3B (Abmart, T55992, 1:1000), Atg10 (Abmart, TD8366, 1:1000), GAPDH (Transgen, HC301-02, 1:1000), then incubated menbrane with HRP-conjugated secondary antibody (Beyotime, 1:1000). (The brands and dilution ratios of the antibodies used are detailed in Additional file 1: Table S4). An enhanced chemiluminescence assay (Tanon, 180-506) was used to visualize the protein (Tanon, 4600). Immunoreactivity were analyzed using ImageJ.

### Blood total cholesterol levels

Blood samples were obtained by tail amputation and serum was obtained after centrifugation. Total cholesterol was measured by Automated Chemistry Analyzer (Chemray 240), according to CHOD-PAP method.

### Statistical analysis

The comparisons of two independent data points were analyzed using the unpaired two-tailed t-test. Statistical significance analysis was performed using GraphPad Prism, expressed as *p < 0.05, **p < 0.01, ***p < 0.001, ****p < 0.0001.

### Supplementary Information

**Additional file 1: Figure S1.** The diabetic model was successfully constructed after STZ injection. **Figure S2.** Internalization of plasma exosomes of sciatic nerve in NC rats. **Figure S3.** Biological distribution of plasma exosomes in vivo. **Figure S4.** Effects of plasma exosomes on RSC96 and DRG cells. **Figure S5.** Effects of plasma exosomes on RSC96 and DRG cells. **Figure S6.** Statistical analysis of pstat3/stat3. **Figure S7.** Characterization of ageing plasma exosomes. **Figure S8.** Ageing-exos did not improve nerve damage caused by high glucose. **Figure S9.** Ageing-exos augments the motor and sensory innervation of the targets. **Table S1.** Random blood glucose and total cholesterol levels after exosome treatment. **Table S2.** Random blood glucose and total cholesterol levels after miR-20b-3p agomir treatment. **Table S3.** Sequence information used in the article. **Table S4.** Reagent information used in the article.

## Data Availability

Data sets used and/or analyzed during the current study are available from first authors upon reasonable request.
