# Supplementary material for: Plasma exosomes improve peripheral neuropathy via miR-20b-3p/Stat3 in type I diabetic rats
Source: J Nanobiotechnology. 2023 Nov 24;21:447. doi: 10.1186/s12951-023-02222-5 (PMC10675980; doi:10.1186/s12951-023-02222-5)
Supplement: Supplementary file 1 — Additional file 1: Figure S1. The diabetic model was successfully constructed after STZ injection. Figure S2. Internalization of plasma exosomes of sciatic nerve in NC rats. Figure S3. Biological distribution of plasma exosomes in vivo. Figure S4. Effects of plasma exosomes on RSC96 and DRG cells. Figure S5. Effects of plasma exosomes on RSC96 and DRG cells. Figure S6. Statistical analysis of pstat3/stat3. Figure S7. Characterization of ageing plasma exosomes. Figure S8. Ageing-exos did not improve nerve damage caused by high glucose. Figure S9. Ageing-exos augments the motor and sensory innervation of the targets. Table S1. Random blood glucose and total cholesterol levels after exosome treatment. Table S2. Random blood glucose and total cholesterol levels after miR-20b-3p agomir treatment. Table S3. Sequence information used in the article. Table S4. Reagent information used in the article. [file 12951_2023_2222_MOESM1_ESM.docx]

**Additional file 1**

**Plasma exosomes improve peripheral neuropathy via miR-20b-3p/Stat3 in type I diabetic rats**

Jiayang Li^1,2^, Guangzhi Wu^1,2^, Weiye Li^1,2^, Xiongyao Zhou^1,2^, Weizhen Li^1,2^, Xiong Xu^1,2^, Ke Xu^1,2^, Rangjuan Cao^1,2*^, Shusen Cui^1,2^*

1. Department of Hand and Foot Surgery, China-Japan Union Hospital of Jilin University, Changchun, China.

2. Key Laboratory of Peripheral Nerve Injury and Regeneration of Jilin Province, Changchun, China.

**Running title：Plasma exosomes alleviate diabetic peripheral neuropathy**

*** Correspondence**

Shusen Cui, cuiss@jlu.edu.cn.;

Rangjuan Cao, caorj@jlu.edu.cn.

**
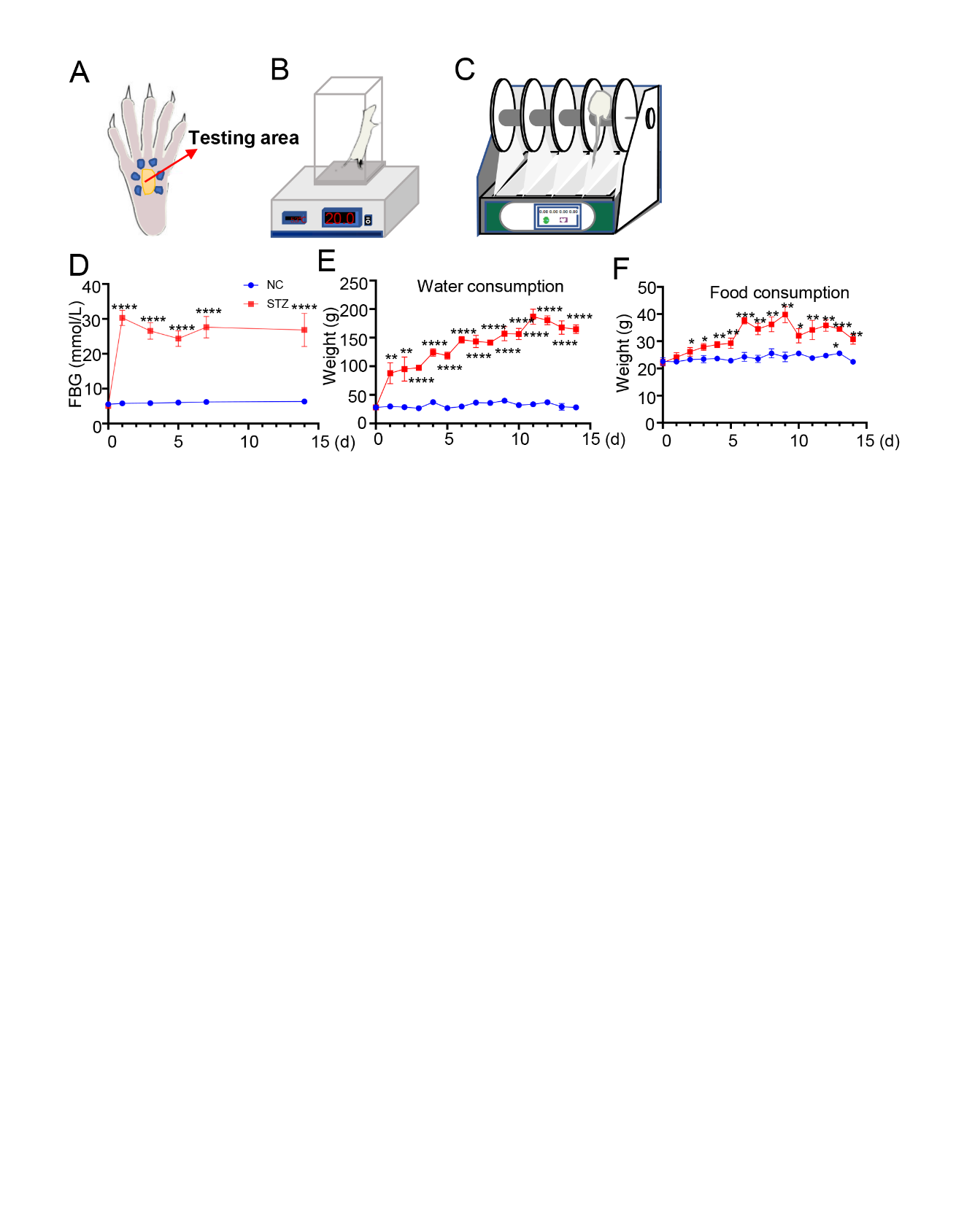
 Figure S1. The diabetic model was successfully constructed after STZ injection**

1. Schematic of Von Frey test. (B) Schematic of plantar test. (C) Schematic of rotarod test. Statistical analysis of random blood glucose changes after STZ injection, n = 7. (E) Statistical analysis of water intake changes after STZ injection, n = 3. (F) Statistical analysis of food intake changes after STZ injection, n = 3. Data are presented as the mean ± SD (*p < 0.05, **p < 0.01, ***p < 0.001, ****p < 0.0001).

**
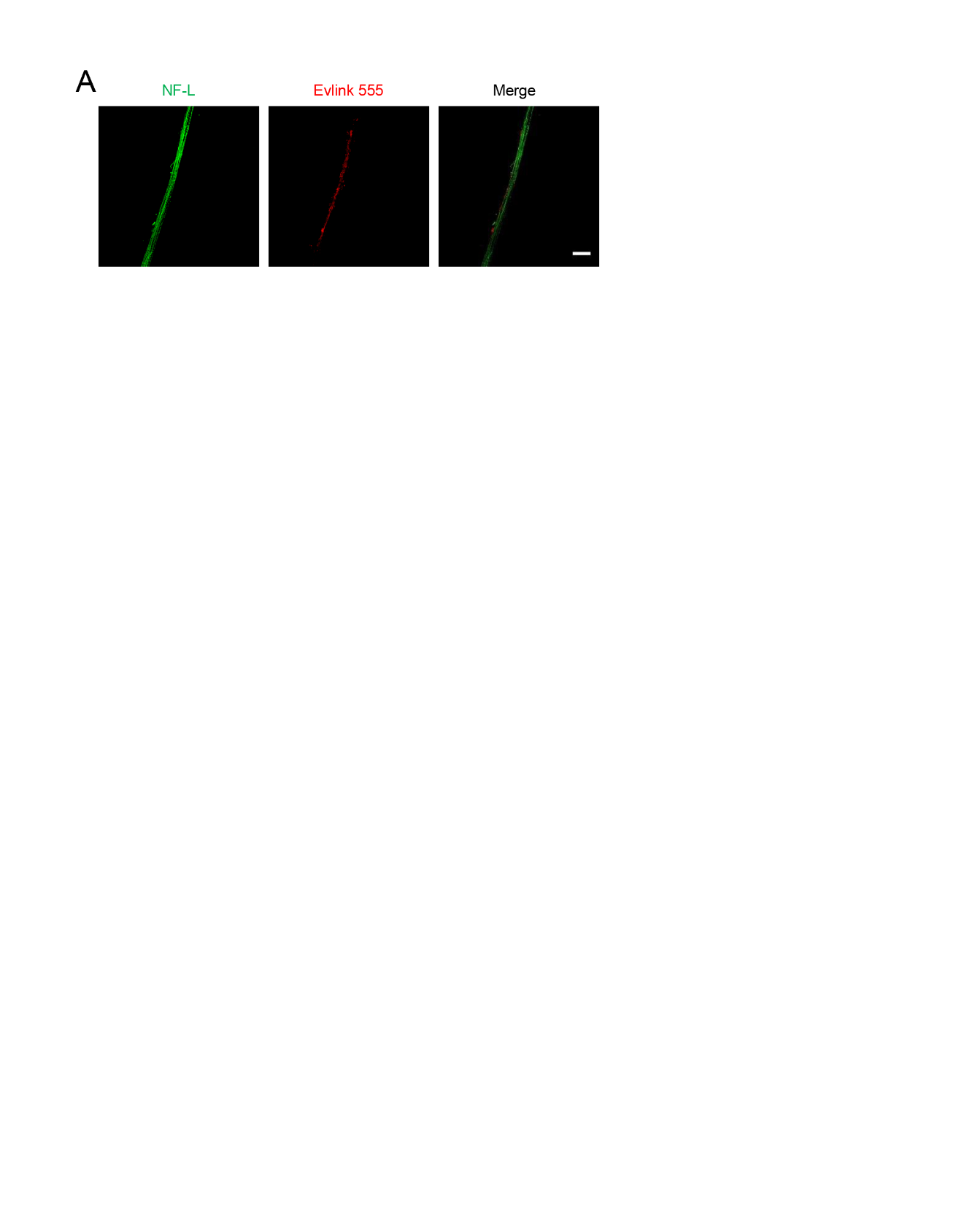
 Figure S2. Internalization of plasma exosomes of sciatic nerve in NC rats**

(A) The red labeled exosomes are internalized by the green labeled sciatic nerve，scale bar = 200 µm.

**
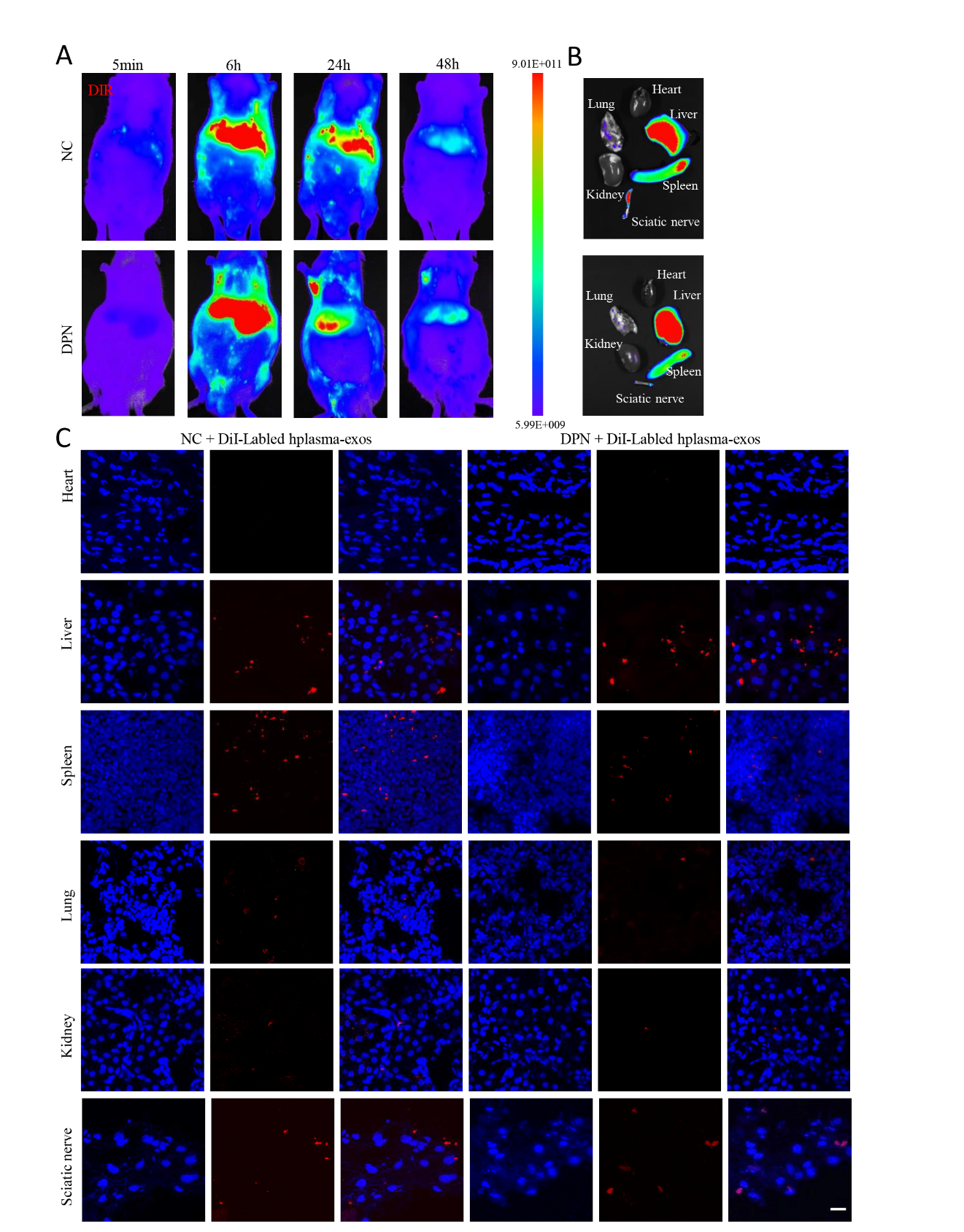
 Figure S3. Biological distribution of plasma exosomes in vivo**

(A) Biological distribution images of NC and DPN rats at different time after injection of DiR-labeled hplasma-exos through the tail vein. (B) The distribution of DiR-labeled hplasma-exos in different groups of rat tissues was detected by IVIS system at 6h after tail vein injection. (C) After 6 hours of injection of DiI-labeled hplasma-exos through the tail vein, different tissues were sampled and stained to observe the distribution of hplasma-exos. Bar = 20 µm.

**
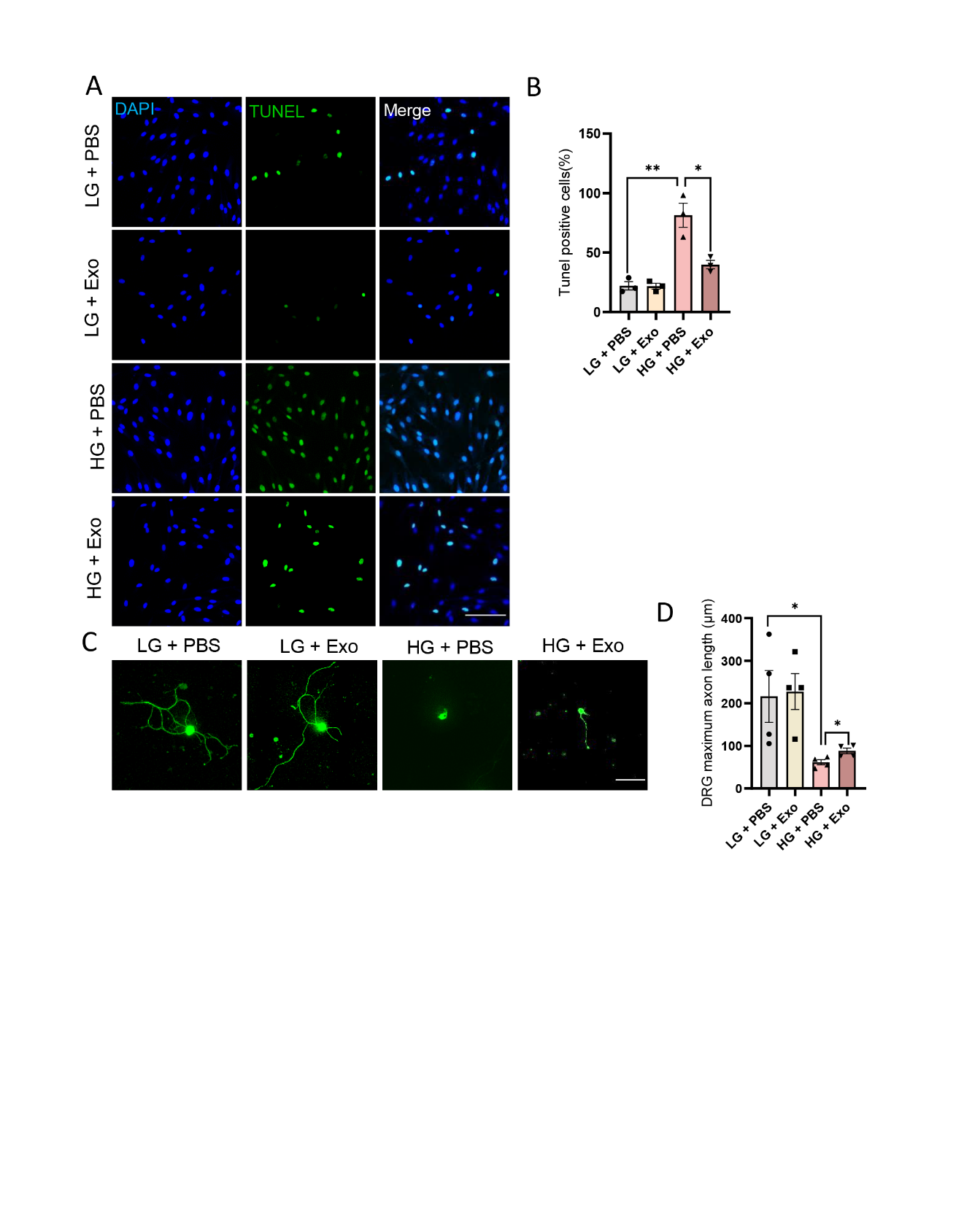
 Figure S4. Effects of plasma exosomes on RSC96 and DRG cells**

(A) Representative images of TUNEL+ RSC96 cells in different groups. scale bar = 100 µm. (B) Statistical results of TUNEL+ cell proportion in differnt groups, n = 3. (C) Representative images of DRG neurons cultured in vitro in different groups of rats. scale bar = 100 µm. (D) Statistical results of the longest axon length of DRG neurons cultured in vitro in different groups, n = 4.

**
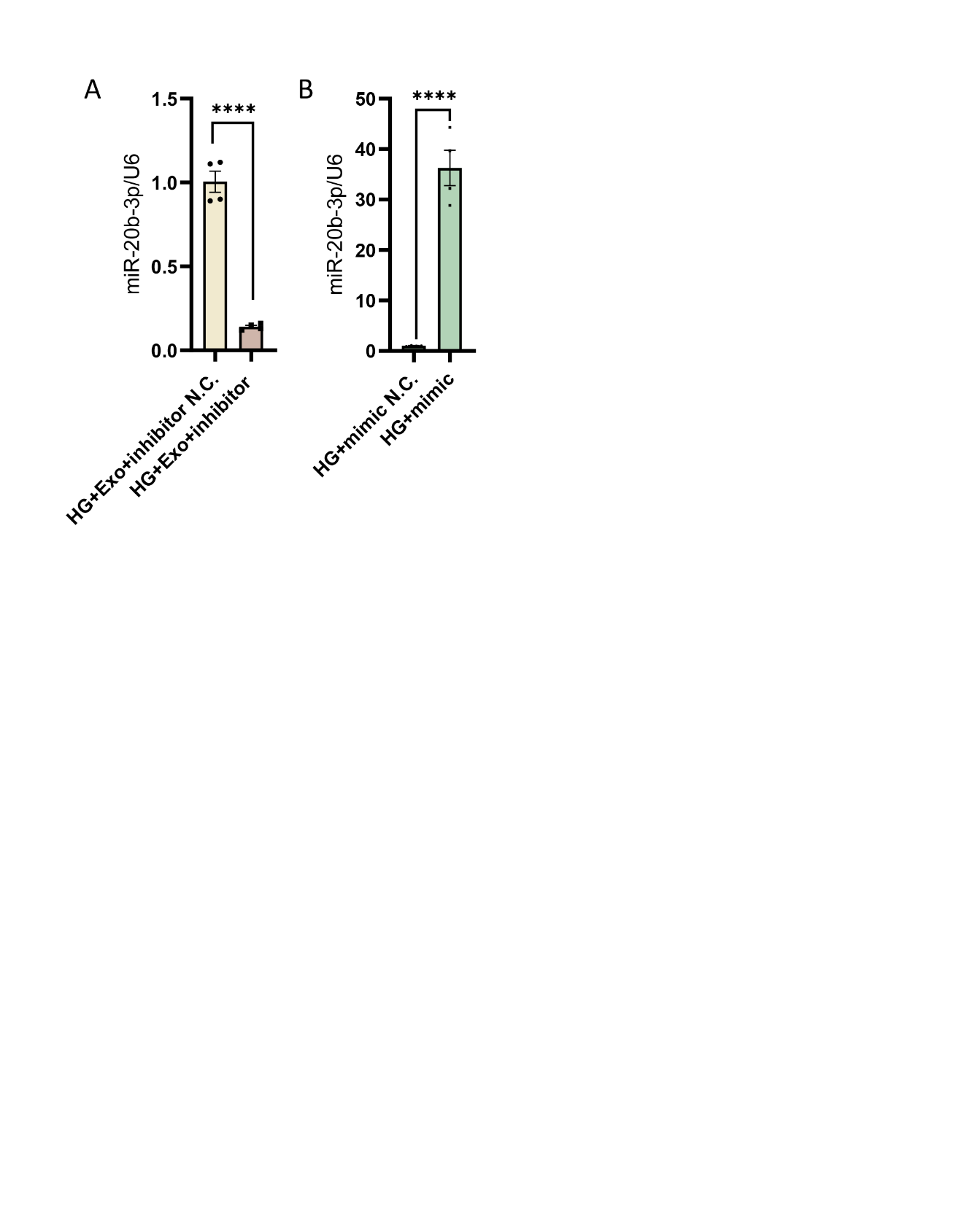
 Figure S5. Effects of plasma exosomes on RSC96 and DRG cells**

(A) Statistical results of the expression of miR-20b-3p after added hplasma-exos and transfection with miR-20b-3p inhibitor N.C. or miR-20b-3p inhibitor, n = 4. (B) Statistical results of expression of miR-20b-3p after transfection with miR-20b-3p stable N.C. or miR-20b-3p mimic, n = 3.

**
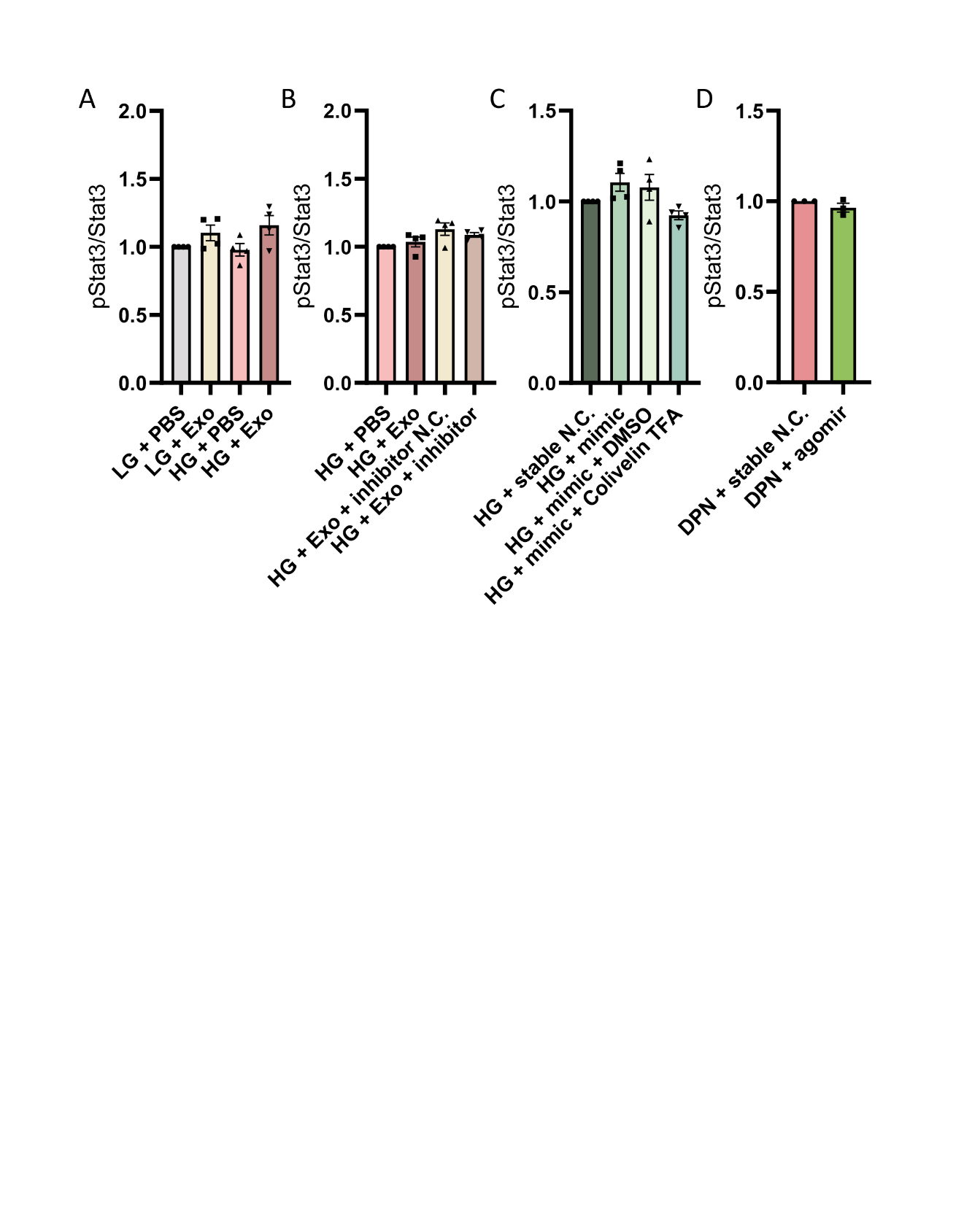
 Figure S6. Statistical analysis of pstat3/stat3**

(A)-(D) Statistical analysis of pstat3/stat3 in RSC96 cells in vitro after different stimulation treatments. n = 3-4.


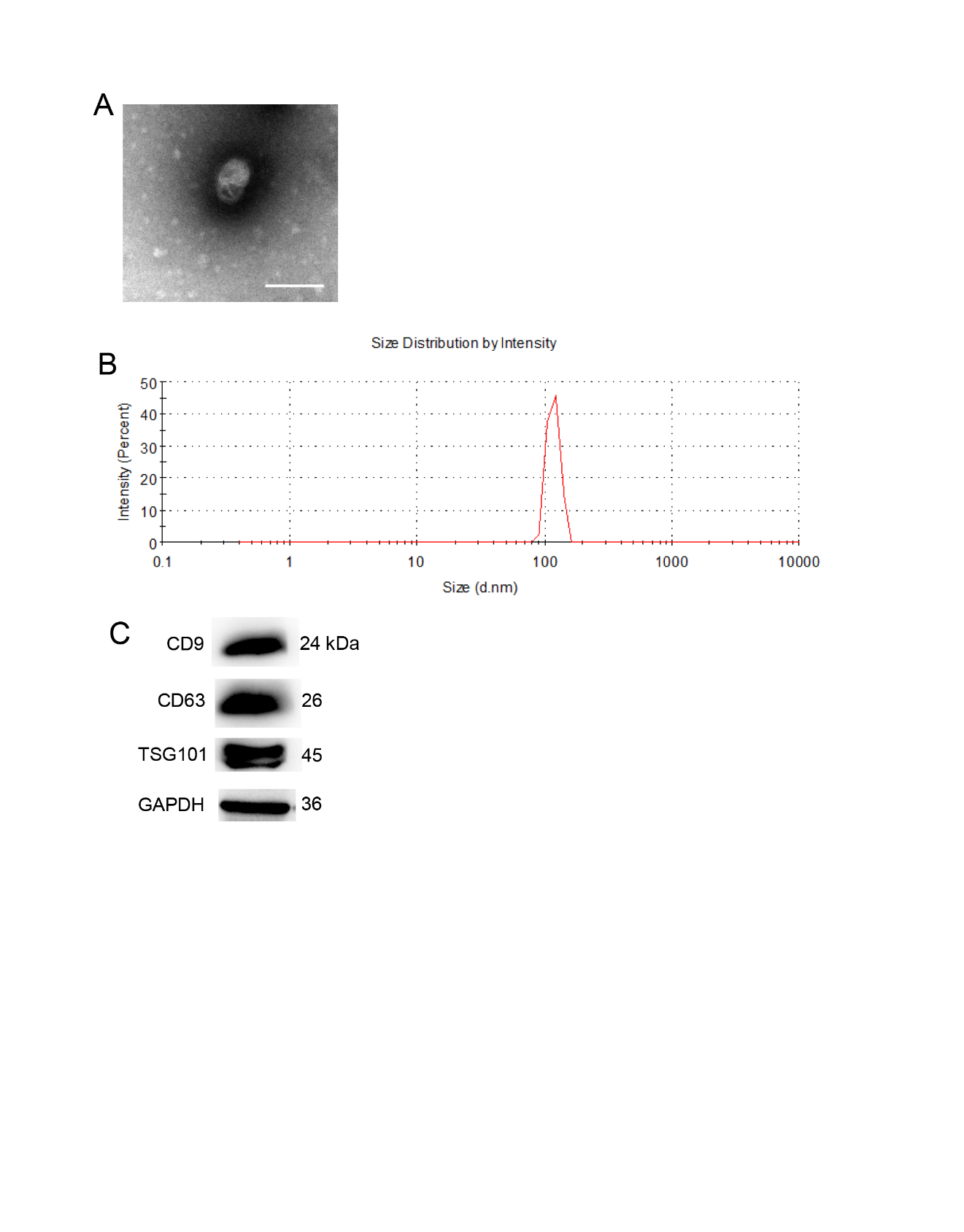


**Figure S7. Characterization of ageing plasma exosomes**

(A) Ageing plasma-derived exosomes characterized by TEM, scale bar = 100 nm. (B) NTA and Western blot (C) were used to characterize the extracted exosomes.

**
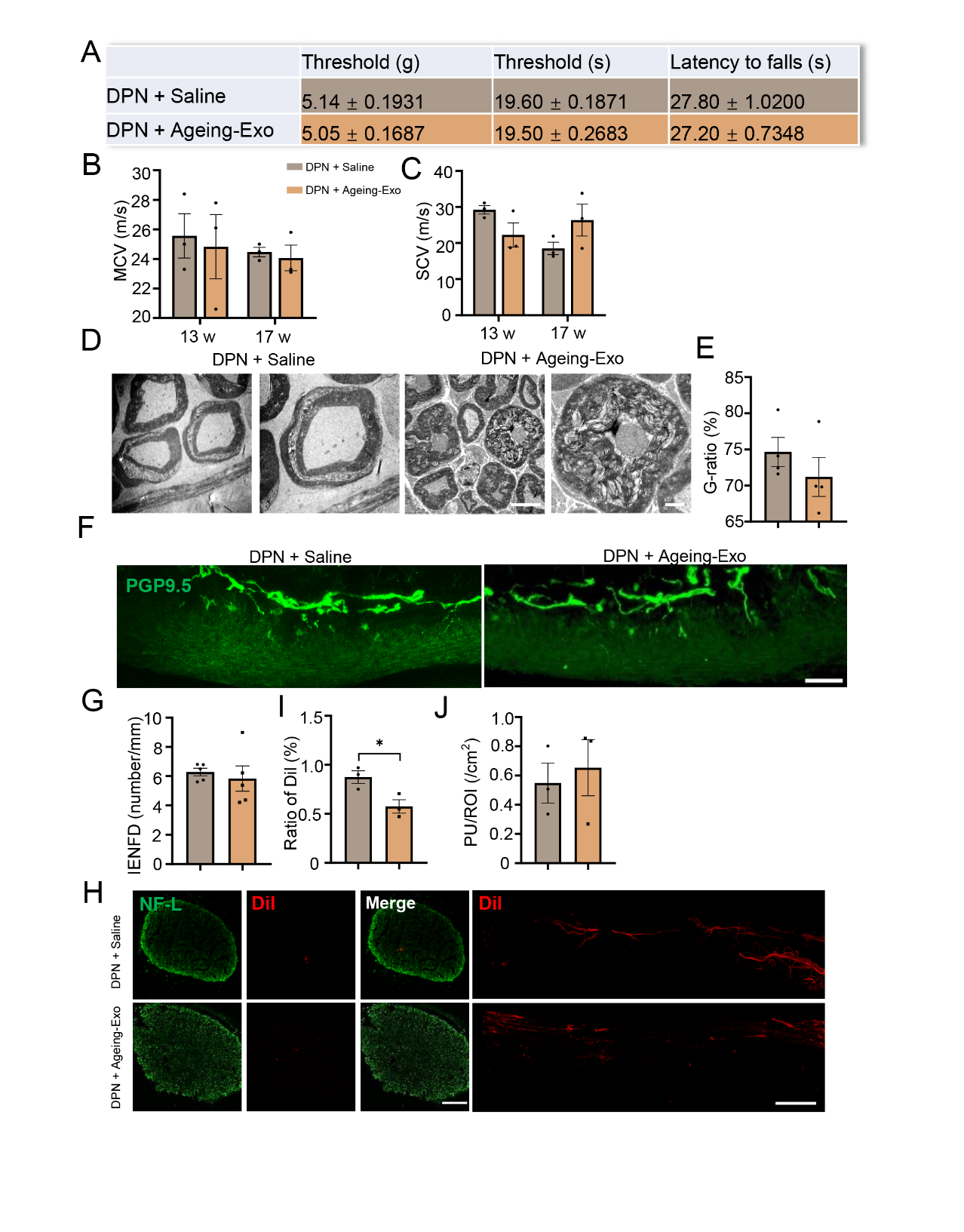
 Figure S8. Ageing-exos did not improve nerve damage caused by high glucose**

1. Statistical analysis of behavioral changes in different groups of rats, n = 5. (B-C) Changes of MCV and SCV after ageing-exos or volume-matched saline treatment, n = 3. (D) The ultrastructure of sciatic nerve was observed by TEM in different groups of rats, scale bar = 5µm/2µm. (E) Histogram represents the quantitative data of the G-ratio under various conditions, n = 4. (F) Representative images showing PGP9.5 positive intraepidermal nerve fibers in the posterior plantar skin of different groups of rats, scale bar = 100 µm. (G) Statistical analysis of IENFD is shown in Figure G, n = 5. (H) Representative images of Dil perfusion in different groups of transverse and longitudinal section. Transverse section, scale bar = 200 µm and longitudinal section, scale bar = 1000 µm. (I) Statistical analysis of Dil (red) fluorescence area ratio in transverse section, n = 3. (J) Statistical analysis of plantar blood perfusion per unit area is shown in figure (I), n = 3. Data are presented as the mean ± SEM. (*p < 0.05, **p < 0.01, ***p < 0.001, ****p < 0.0001).

**
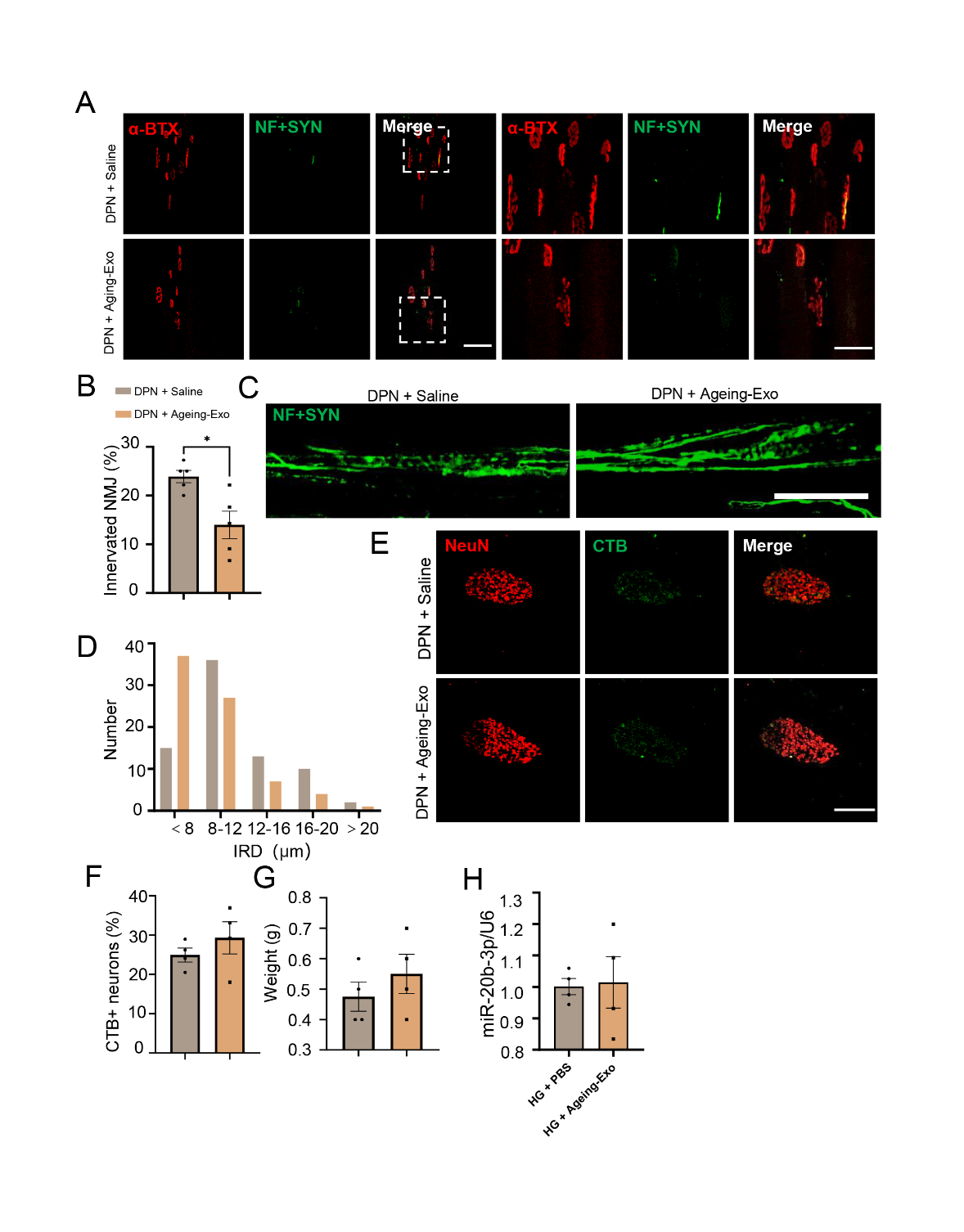
**

**Figure S9. Ageing-exos augments the motor and sensory innervation of the targets**

(A) Representative images of NMJs labeled with 555-a-BTX (red) and NF-L + Syn (green) in different groups, scale bar =100/50 µm. Squares, images enlarged in right panel. (B) Statistical analysis of the proportion of innervated NMJ (partial denervation and total denervation are excluded), n = 5. (C) Representative images of annulospiral endings of muscle spindles in different groups, scale bar = 200 µm. (D) Number of muscle spindles in different IRD distribution intervals. (E) Representative images of different groups of neurons labeled with NeuN (red) and retrograde tracer by FITC-CTB (green), scale bar = 500 µm. (F) Statistical analysis of the proportion of FITC-CTB positive cells, n = 4. (G) Statistical analysis of wet weight of gastrocnemius muscle in each group, n = 4. (H) Statistical results of the expression of miR-20b-3p of RSC96 cultured in vitro after treatment of ageing-exos or the same volume of saline, n = 4. Data are presented as the mean ± SEM. (*p < 0.05, **p < 0.01, ***p < 0.001, ****p < 0.0001).

|  | RBG (mmol/L) | TCH (mmol/L) |
| --- | --- | --- |
| NC + Saline | 5.4 ± 0.1000 | 1.84 ± 0.2205 |
| NC + Exo | 5.3 ± 0.4096 | 1.867 ± 0.1541 |
| DPN + Saline | 27.9 ± 0.7371 | 2.453 ± 0.2216 |
| DPN + Exo | 27.3 ± 0.9504 | 2.469 ± 0.2036 |

Table S1. Random blood glucose and total cholesterol levels after exosome treatment.

|  | RBG (mmol/L) | TCH (mmol/L) |
| --- | --- | --- |
| DPN + Stable N.C. | 27.23 ± 0.5239 | 2.537 ± 0.1497 |
| DPN + Agomir | 27.63 ± 0.4256 | 2.673 ± 0.3690 |

Table S2. Random blood glucose and total cholesterol levels after miR-20b-3p agomir treatment.

|  | **5’-3’ sequence** |
| --- | --- |
| RmiR-20b-3p-FO | TCTTGGGACTGCAGTGTGAGC |
| RmiR-20b-3p-RE | TATGGTTGTTCACGACTCCTTCAC |
| Rno-miR-20b-3p agomir | S:ACUGCAGUGUGAGCACUUCUGG  AS:AGAAGUGCUCACACUGCAGUUU |
| Stable Negative Control | S:UUCUCCGAACGUGUCACGUTT  AS:ACGUGACACGUUCGGAGAATT |
| Rno-miR-20b-3p mimics | S:ACUGCAGUGUGAGCACUUCUGG  AS:AGAAGUGCUCACACUGCAGUUU |
| Rno-miR-20b-3p inhibitor | CCAGAAGUGCUCACACUGCAGU |
| Rno-miR-20b-3p inhibitor N.C. | CAGUACUUUUGUGUAGUACAA |

Table S3. Sequence information used in the article.

| **Reagent**  **name** | **Reagent brand** | **Working Concentration** | **Reagent**  **name** | **Reagent brand** | **Working Concentration** |
| --- | --- | --- | --- | --- | --- |
| CD9 | Santa  SC13118 | 1:200 | CD63 | Santa  SC5275 | 1:200 |
| TSG101 | Santa  SC7964 | 1:200 | Neurofilament-L  (NF-L) | CST C28E10 | 1:300 |
| S100β | Sigma  S2532 | 1:500 | PGP9.5 | R&D  MAB60072 | 1:200 |
| Synapsin-1  (Syn) | CST  5297S | 1:800 | α-bungarotoxin  (α-BTX) | Invitrogen  CF568 | 1:1000 |
| NeuN | CST  24307S | 1:500 | DAPI | Genview  GD3410 | 1:1000 |
| FITC  conjugated (CTB) | Absin  abs80003 | 0.4 mg/ml | GAPDH | Transgen  HC301-02 | 1:1000 |
| STAT3 | Abmart  T55292 | 1:1000 | Phospho-STAT3(pSTAT3) | Abmart  T56566 | 1:1000 |
| LC3B | Abmart  T55992 | 1:1000 | Atg10 | Abmart  TD8366 | 1:1000 |
| Alexa Flour 546 Donkey AntiRabbit IgG | Invitrogen  A10040 | 1:500 | Alexa Flour 488 Donkey AntiMouse IgG | Invitrogen  A21202 | 1:500 |
| Alexa Flour 546Donkey AntiMouse IgG | Invitrogen  A11030 | 1:500 | Alexa Flour 488Donkey AntiRabbit IgG | Invitrogen  A11008 | 1:500 |
| HRP-labeled Goat Anti-Mouse IgG | Beyotime  A0216 | 1:1000 | HRP-labeled Goat Anti-Rabbit IgG | Beyotime  A0208 | 1:1000 |

Table S4. Reagent information used in the article.
